# Insights into bacterial cell division from a structure of EnvC bound to the FtsX periplasmic domain

**DOI:** 10.1073/pnas.2017134117

**Published:** 2020-10-23

**Authors:** Jonathan Cook, Tyler C. Baverstock, Martin B. L. McAndrew, Phillip J. Stansfeld, David I. Roper, Allister Crow

**Affiliations:** ^a^School of Life Sciences, University of Warwick, Coventry, CV4 7AL, United Kingdom;; ^b^Warwick Medical School, University of Warwick, Coventry, CV4 7AL, United Kingdom;; ^c^Department of Chemistry, University of Warwick, Coventry, CV4 7AL, United Kingdom

**Keywords:** bacterial cell division, peptidoglycan, ABC transporters, X-ray crystallography

## Abstract

FtsEX is a bacterial ABC transporter that regulates the activity of periplasmic peptidoglycan amidases via its interaction with the murein hydrolase activator, EnvC. In *Escherichia coli*, FtsEX is required to separate daughter cells after cell division and for viability in low-osmolarity media. Both the ATPase activity of FtsEX and its periplasmic interaction with EnvC are required for amidase activation, but the process itself is poorly understood. Here we present the 2.1 Å structure of the FtsX periplasmic domain in complex with its periplasmic partner, EnvC. The EnvC-FtsX periplasmic domain complex has a 1-to-2 stoichiometry with two distinct FtsX-binding sites located within an antiparallel coiled coil domain of EnvC. Residues involved in amidase activation map to a previously identified groove in the EnvC LytM domain that is here found to be occluded by a “restraining arm” suggesting a self-inhibition mechanism. Mutational analysis, combined with bacterial two-hybrid screens and in vivo functional assays, verifies the FtsEX residues required for EnvC binding and experimentally test a proposed mechanism for amidase activation. We also define a predicted link between FtsEX and integrity of the outer membrane. Both the ATPase activity of FtsEX and its periplasmic interaction with EnvC are required for resistance to membrane-attacking antibiotics and detergents to which *E. coli* would usually be considered intrinsically resistant. These structural and functional data provide compelling mechanistic insight into FtsEX-mediated regulation of EnvC and its downstream control of periplasmic peptidoglycan amidases.

## Significance

The peptidoglycan layer is a core component of the bacterial cell envelope that provides a barrier to the environment and protection from osmotic shock. During division, bacteria must break and rebuild the peptidoglycan layer to enable separation of daughter cells. In *E. coli*, two of the three amidases responsible (AmiA and AmiB) are regulated by a single periplasmic activator (EnvC) that is, itself, controlled by an atypical ABC transporter (FtsEX) tethered to the cytoplasmic septal Z-ring. Here we define the structural basis for the interaction of FtsEX with EnvC and suggest a molecular mechanism for amidase activation where EnvC autoinhibition is relieved by ATP-driven conformational changes transmitted through the FtsEX-EnvC complex.

FtsEX is a type VII ABC transporter (1, 2) belonging to the ABC3 superfamily (3). Distant relatives of FtsEX include the MacB efflux pump (1), LolCDE lipoprotein trafficking machinery (1, 4), PvdT pyoverdine recycling system (5), and BceAB family of antibiotic resistance proteins (6). The best-characterized example is MacB which serves as a structural archetype for the type VII ABC transporter superfamily (1). Multiple structures of MacB are known (1, 7–9) and a comparison of ATP-bound and nucleotide-free structures has revealed a distinctive mechanotransmission mechanism in which cytoplasmic ATP binding and hydrolysis drive the periplasmic domains to perform work on the opposite face of the membrane (1). The mechanotransmission mechanism is tightly wed to the structural architecture of type VII ABC transporters and it is anticipated that similar conformational changes are conserved throughout this superfamily including FtsEX (1, 2, 10).

FtsEX is a transmembrane signaling complex that coordinates periplasmic peptidoglycan remodelling with cytoplasmic cell division events (10–12). Central to this role is the ability of FtsEX to regulate periplasmic amidases via its large periplasmic domain (located between the first and second transmembrane helix of FtsX) (10–12). In the early stages of bacterial cell division, FtsEX is recruited to midcell via its interaction with filament-forming FtsA and FtsZ proteins in the cytoplasmic Z-ring (10, 13). The FtsEX ATP-binding and -hydrolysis cycle then coordinate long-range conformational changes that activate amidases on the opposite face of the membrane (11, 12, 14). Peptidoglycan amidase activity is required to break the peptidoglycan sacculus and enable daughter cell separation. In gram-positive organisms such as *Streptococcus* *pneumoniae*, FtsEX interacts directly with peptidoglycan hydrolases such as PcsB (15, 16), but in *Escherichia coli*, and other gram-negative bacteria, FtsEX interacts with a periplasmic intermediary, EnvC, that activates downstream amidases (12). EnvC is termed a “murein hydrolase activator” and has been shown to stimulate peptidoglycan hydrolysis by both AmiA and AmiB in vitro (17). A structure of the C-terminal LytM domain of EnvC has revealed a likely binding site for AmiA and AmiB (18), but how EnvC’s activating function is regulated by FtsEX remains unclear.

In *E. coli*, strains lacking FtsEX fail to segregate daughter cells after division giving rise to a chaining phenotype in which cells are joined to one another by a continuous peptidoglycan layer and grow poorly on low-osmolarity media (19, 20). The ATPase activity of FtsEX is required for both daughter cell segregation and low-salt viability, but only the first of these functions strictly requires the interaction with EnvC (13). The chaining phenotype is linked directly to FtsEX’s role in activating periplasmic amidases, while low-salt viability probably stems from a secondary function of FtsEX in recruiting cell division proteins to the division site (13, 14). FtsEX is also predicted to have a role in maintaining outer membrane integrity since EnvC and amidase-deficient strains show cell envelope defects in several organisms (21–25).

Here we present the crystal structure of *E. coli* EnvC in its entirety, bound to the periplasmic domains of FtsX. Using the structure, we define a detailed molecular description of the FtsEX-EnvC interaction and uncover an autoinhibition mechanism at the level of EnvC. We also demonstrate a role for FtsEX in maintaining intrinsic resistance to antibiotics and detergents that depends on both its ATPase activity and periplasmic interaction with EnvC. The data support a molecular mechanism for amidase control where mechanotransmission-driven conformational change in FtsEX is propagated through the EnvC coiled coil domain to release an autoinhibitory element from within its LytM domain. These results have important implications for understanding FtsEX function in both cell division and outer membrane integrity.

## Results

### A 2.1 Å Crystal Structure of EnvC Bound to the FtsX Periplasmic Domain.

**Fig. 1.**
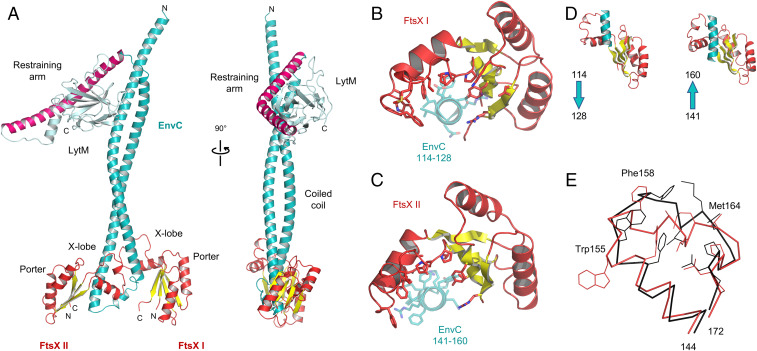
Structure of EnvC bound to the FtsX periplasmic domains. (*A*) Complete structure of mature *E. coli* EnvC bound to two molecules of the FtsX periplasmic domain. The two orientations shown are related by a 90° rotation about the vertical axis. (*B*) Close-up view of the interaction between the FtsX periplasmic domain and the first helix of the EnvC coiled coil domain. (*C*) An equivalent view of the second FtsX monomer bound to the second coiled coil helix. Interacting sidechains are shown with stick representation, and the bound helical elements of EnvC are partially transparent. (*D*) Side-by-side views of the FtsX periplasmic domains with bound portions of EnvC. Arrows indicate the directionality of EnvC helix. (*E*) Comparison of the X-loop conformation (residues 144 to 172 of FtsX) for the two EnvC-bound FtsX periplasmic domain monomers. FtsX I in red, and FtsX II in black. Sidechains of residues Phe152, Trp155, Phe158, Ala161, Leu162, Met164, Leu165, Pro169, Leu170, and Pro171 are shown as thin lines. Trp155, Phe158, and Met164 are explicitly labeled because they exhibit particularly large conformational differences.

We determined the structure of mature EnvC in complex with the FtsX periplasmic domain using X-ray crystallography. The structure is shown in Fig. 1*A* with X-ray data and refinement statistics reported in *SI Appendix*, Table S1. A movie documenting the quality of the underpinning electron density is given as *SI Appendix* (Movie S1). Crystals of the EnvC-FtsX periplasmic domain complex belong to space group P2_1_2_1_2_1_ with two molecules of the FtsX periplasmic domain and one molecule of EnvC in the asymmetric unit. Analysis of protein contacts using PISA (26) shows that the observed 2-to-1 complex is highly stable and therefore likely to be biologically relevant.

EnvC has a tripartite structure with three distinct functional domains: A globular C-terminal LytM domain comprising the proposed amidase activation site (Fig. 1*A* cyan), a central regulatory domain (Fig. 1*A* pink), and an N-terminal FtsEX-interacting coiled coil domain (Fig. 1*A* teal) that is here bound to two periplasmic domain modules of FtsX (Fig. 1*A* red/yellow). A structure of the C-terminal LytM domain has been described previously (18), but both the N-terminal FtsEX-binding domain and central regulatory domain (which we term the “restraining arm”) are additionally characterized here. Similarly, structures of the FtsX periplasmic domain are known (11, 27), but these are distinct from the complex presented here showing FtsX engaged to its periplasmic partner.

### Description of the EnvC C-Terminal Domain and Regulatory Domain.

The C-terminal LytM domain of EnvC has an endopeptidase-like fold belonging to the M23 peptidase family (18). The LytM domain lacks catalytic residues necessary for peptidoglycan hydrolase activity (18) but has been shown to activate amidases in vitro (17). A groove in the surface of the LytM domain forms the amidase recruitment site (18) which is here found to be blocked by the regulatory domain (Fig. 1*A*). The regulatory domain (which we term the restraining arm) is composed of a 50-residue α-helix with a 70° kink midway along its length (Fig. 1 *A*, *Right*). Occupation of the amidase-binding site by the restraining arm strongly suggests that EnvC has been captured in an autoinhibited state that would necessitate a conformational change in order to bind and activate its cognate amidases.

### Description of the EnvC Coiled Coil Domain.

The N-terminal coiled coil domain of EnvC has a hairpin-like structure formed by a pair of antiparallel coiled coil helices (long helices I and II) that are joined by a short linker (Fig. 1*A*). The linker contains a six-residue helix flanked on either side by regions of extended peptide. Inspection of the amino acid conservation within the N-terminal domain shows 25 seven-residue “heptad” repeats that are conserved among EnvC homologs (*SI Appendix*, Fig. S1*A*). The heptad repeats fall within two stretches on either side of the linker and are clearly important for formation of the coiled coil. We analyzed the composition of the heptad repeats (*SI Appendix*, Fig. S1*B*) and their positions within the EnvC structure (*SI Appendix*, Fig. S1 *C* and *D*). The first residue of each heptad is typically a leucine, isoleucine, or valine, and the fourth and fifth positions are most often occupied by either leucine or glutamine (*SI Appendix*, Fig. S1*B*). EnvC has 12 heptad repeats within long helix I (heptads 1 to 12; residues 40 to 123) and another 9 heptads within long helix II (heptads 13 to 21; residues 152 to 214). Consistent with formation of the antiparallel coiled coil, the first, fourth, and fifth positions of each heptad mediate interhelix contacts through classical knobs-into-holes packing (28)—the first residue of each heptad forming a “knob” and the fourth and fifth forming a “hole.” The EnvC coiled coil is slightly overwound in comparison to a coiled coil of “ideal” geometry, suggesting that it may be under strain.

The 25 heptads are shown schematically in *SI Appendix*, Fig. S1*C* and mapped to the EnvC structure in *SI Appendix*, Fig. S1*D*. Heptads 4 to 12 pair with heptads 14 to 21 in the coil, but heptads 1 to 3 and 22 to 25 are unpaired. Heptads 1 to 3 are located at the EnvC N-terminus and face the solvent while heptads 23 to 25 are located within the restraining arm facing toward the interior of the LytM domain (*SI Appendix*, Fig. S1 *C* and *D*). Heptad 22 forms a linker that joins the coiled coil to the restraining arm. The unpaired heptad repeats found within the EnvC N-terminus and the restraining arm are both highly unusual and well conserved, suggesting they are mechanistically important.

### Description of the *E. coli* FtsX Periplasmic Domains.

The two FtsX periplasmic domains bound to EnvC are structurally similar to one another and can be superposed with an rmsd of 1.5 Å^2^. The FtsX monomer bound to the more N-terminal portion of EnvC (long helix I) is well ordered with B-factors that are comparable to those of EnvC. The second monomer, which is bound to long helix II, has higher B-factors, indicating increased mobility—particularly for the first 40 residues. Nonetheless, FtsX residues contacting EnvC are properly resolved in the electron density for both FtsX monomers, giving confidence in the binding site structure and assigned stoichiometry (Movie S1).

The folds of the two EnvC-bound *E. coli* FtsX fragments are similar to those of *Mycobacterium tuberculosis* (11) and *S. pneumoniae* proteins (27) for which structures have been determined in isolation (*SI Appendix*, Fig. S2). The FtsX periplasmic domain comprises two βαβ secondary structure motifs that interlock to form a central four-membered β-sheet with two flanking helices. This domain is homologous with the Porter domain found in MacB and LolC and is a conserved feature of all type VII ABC transporters (1, 2). A prominent pair of protruding helices, termed the X-lobe (10), is located between the two βαβ motifs where the Sabre domain is located in MacB and LolC (1). The presence of the X-lobe in FtsX and its absence from other type VII ABC transporters signals its importance for FtsX-specific functions, and indeed, the X-lobe has previously been implicated in EnvC binding (12, 13, 27). The structure presented here confirms the X-lobe as the site of EnvC binding and reveals fine details of the FtsX-EnvC interface.

### Structural Basis for EnvC Binding by the FtsX Periplasmic Domains.

The two FtsX-EnvC interfaces are shown in Fig. 1 *D* and *E* with further detailed views in Movie S2. The first interface is located at the C-terminal end of the first long helix of the coiled coil domain and is composed of EnvC residues 114 to 128. The second interface is located at the N-terminal end of the second long helix and is composed of EnvC residues 141 to 160. The two interfaces are mostly hydrophobic and dominated by interactions arising from the X-lobe (residues 145 to 171) which partially wraps around each of the two long helices that make up the EnvC coiled coil (Fig. 1 *D* and *E*). Prominent interface residues in the X-lobe include Phe152, Phe158, Leu162, and Leu165 which make multiple contacts with EnvC. Phe158 is notable for its position at the tip of the X-lobe where it slots between the two antiparallel helices of EnvC, locking each FtsX domain in place. Additional interactions arising from beyond the X-lobe include a cluster of sidechains (Tyr114, Thr112, Val173, and Val175) that are located on the central β-sheet of the FtsX Porter domain. There are very few hydrogen bonds (PISA detects four in each interface, with areas of 1,134 Å^2^ and 932 Å^2^) and salt bridges are only found at the periphery of each binding surface (FtsX Lys117-EnvC Asp122 and Asp202-Arg126 in the first, Glu151-Arg147 in the second). Association between FtsX and EnvC appears to be driven, primarily, by hydrophobic interactions between complementary surfaces formed as EnvC coiled coil helices slot between the X-lobe and central β-sheets of the FtsX periplasmic domains.

### Dual Recognition of the EnvC Coiled Coil Domain by FtsX.

The set of FtsX residues contacting the two EnvC-binding sites are nearly identical for both interfaces, suggesting the same molecular surface recognizes two structurally distinct target sites—an example of dual recognition. Each FtsX monomer recognizes a helical section of the coiled coil, but the helices themselves are orientated in different directions with respect to the periplasmic domain interface (Fig. 1*D*). Flexibility in the X-lobe is crucial for dual recognition since the X-lobe has to adopt different conformations in each interface to accommodate two different binding surfaces (Fig. 1*E*). The sidechains of Phe158 and Trp155 are particularly mobile with conformational differences on the order of 4.7 Å and 12.7 Å, respectively. The equivalent residues of *S. pneumoniae* FtsX are also perturbed by PcsB binding in NMR experiments (27). Trp155 is notable for forming two very different interactions in each interface—in the first, Trp155 is buried in a hydrophobic nook formed by the aliphatic portions of EnvC residues Ala124, His130, and Glu129, while in the second monomer, it is solvent exposed and forms a cation pi-stack (29) with Arg144 of EnvC. Similarly, FtsX-Glu151 forms a salt bridge with EnvC-Arg147 in one interface, but is completely solvent exposed in the other. Examples such as these are exceptional, however, and most FtsX-EnvC interactions involve hydrophobic interactions that are readily substituted for one another between interfaces. The biological consequence of dual recognition is that a single, internally asymmetric, molecule of EnvC is able to bind to two identical molecules of FtsX. A flexible hydrophobic interface in FtsX facilitates dual recognition of both sites using a single molecular surface.

### Dissection of the EnvC-Binding Site of FtsEX.

**Fig. 2.**
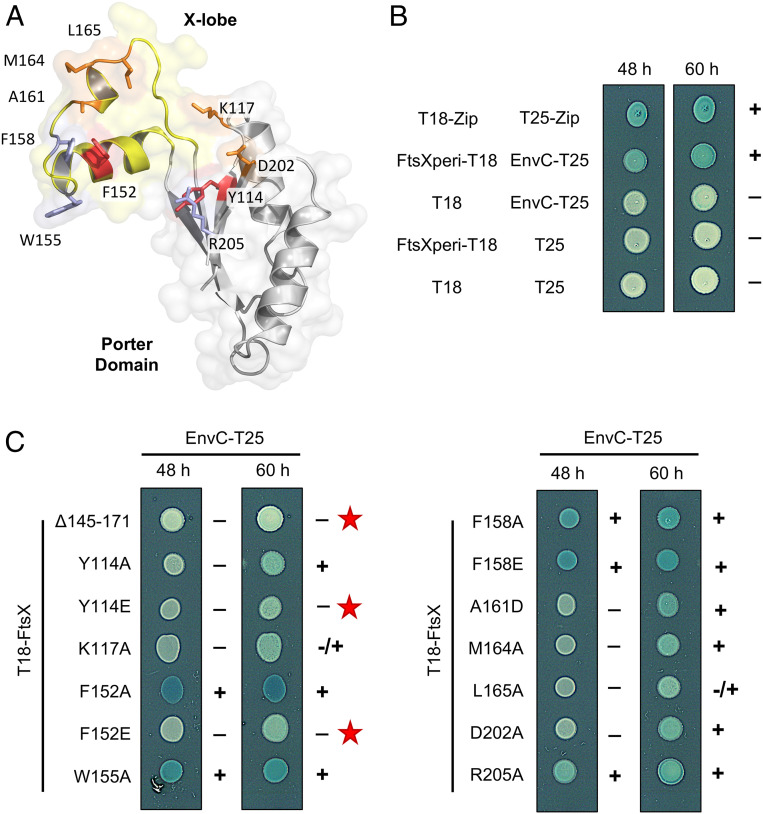
Mutations in the FtsX periplasmic domain impair EnvC binding. (*A*) Location of interface residues in FtsX targeted for mutagenesis. The FtsX Porter domain is shown in gray, with the X-lobe in yellow. Positions of single-site mutants are shown in red, orange, or blue in accordance to whether they disrupt EnvC-binding strongly, modestly, or not at all. (Note that deletion of the X-lobe also completely abrogates binding.) (*B*) Bacterial two-hybrid interaction for the FtsX periplasmic domain construct (FtsX residues 110 to 209) and EnvC (35 to 419). The T18-Zip/T25-Zip interaction is a standard positive control for the BACTH system. (*C*) Bacterial two-hybrid for indicated T18-FtsX variants (full-length FtsX numbering) with EnvC-T25. The Δ145-171 variant lacks the entire X-lobe. Red stars indicate the three variants that were most delayed in coloration over the time course.

To validate the FtsX-EnvC interface and identify variants that can be used to probe the role of FtsEX-EnvC in amidase activation, we made single amino acid substitutions in the FtsX periplasmic domain and tested their interactions with EnvC using the bacterial two-hybrid assay. Guided by structure, we made 13 individual amino acid substitutions at 10 separate positions in the EnvC-binding interface of FtsX (Fig. 2*A*). We also made a substitution variant in which the X-lobe (residues 145 to 171) was replaced by a pair of glycines (hereon referred to as Δ145-171). The Δ145-171 variant is similar to a 152 to 161 deletion mutant previously shown to break binding of EnvC to FtsX (13), but encompasses the complete EnvC-binding loop elucidated by the crystal structure. A bacterial two-hybrid experiment probing interaction of the FtsX periplasmic domain (residues 110 to 209) with EnvC (35 to 419) is shown in Fig. 2*B* alongside relevant positive and negative controls. Further bacterial two-hybrid results obtained with the FtsX variants are shown in Fig. 2*C*. A strong interaction was observed for the wild-type (WT) FtsX periplasmic domain and F152A, W155A, F158A, F158E, and R205A substitutions. However, Y114A, Y114E, K117A, F152E, A161D, M164A, L165A, and D202A variants were all delayed in coloration, suggesting impaired EnvC binding. Only the Δ145-171 mutation completely abrogated binding, although the F152E point variant was significantly impaired. These results confirm the binding interface observed by X-ray crystallography and highlight the Δ145-171 and F152E variants as tools for further assessing the importance of the FtsX-EnvC interface in vivo.

### FtsEX Variants Impaired in EnvC Binding Support Growth on Low-Osmolarity Media but Not Daughter Cell Separation.

We next characterized our series of interface mutants in the context of full-length FtsEX. We first tested each variants’ ability to support growth on low-osmolarity media since this is an established phenotype associated with FtsEX deficiency (19) and a useful check on the integrity of the periplasmic domain mutants (13). Using the well-characterized *E. coli* strains, MR2 (wild type) (19) and MR10 (lacking *ftsEX* due to insertion of a kanamycin cassette) (19), we established a plasmid-based complementation system where different FtsEX variants could be tested. As expected, strains carrying wild-type *ftsEX* grow well, but those for which *ftsEX* is absent, or dependent on an FtsEX variant predicted to have impaired ATPase activity (FtsE K41A, E163Q), do not (*SI Appendix*, Fig. S3*A*). We then tested the periplasmic domain variants, knowing that the EnvC-binding function of FtsEX is dispensable for correcting the low-salt growth defect (13). All interface variants and the X-lobe deletion strain complement *ftsEX* deficiency to wild-type levels, confirming their expression and integrity (*SI Appendix*, Fig. S3*B*). Similar results were obtained for growth in low-salt broth, although FtsEX variants impaired in EnvC binding grew slower than the wild type in a pattern that broadly matched the degree to which binding was impaired (*SI Appendix*, Fig. S3*C*). The results confirm that all 13 FtsEX periplasmic domain variants, including F152E and the X-lobe deletion (Δ145-171), are folded and expressed well enough to rescue growth on low-osmolarity media regardless of whether or not they can bind EnvC.

**Fig. 3.**
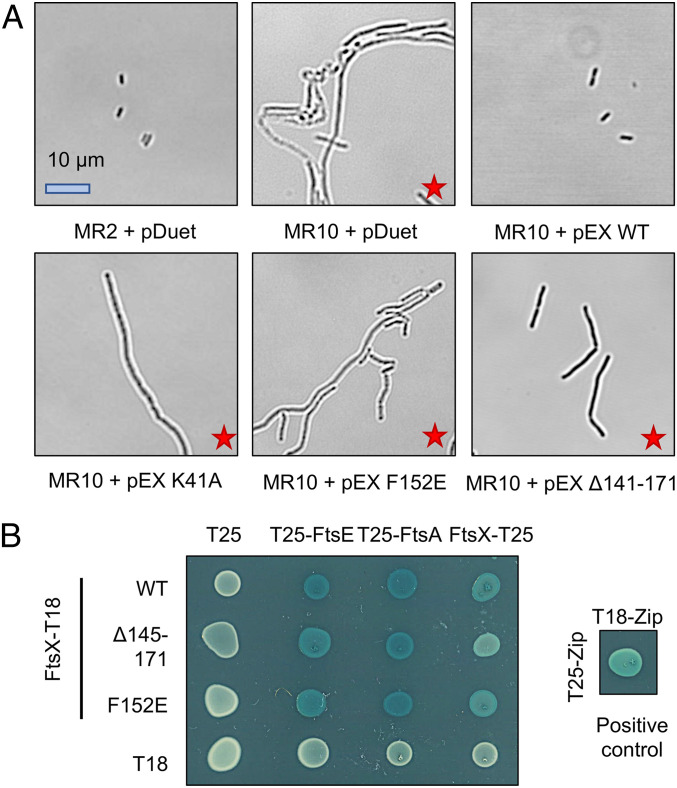
FtsEX variants that are impaired in EnvC binding or ATP hydrolysis fail to separate daughter cells after division. (*A*) Wild-type (MR2) or *ftsEX*-deficient (MR10) *E. coli* complemented with either an empty vector (pDuet) or an FtsEX variant (pEX). pEX WT indicates wild-type FtsEX; pEX K41A indicates an ATPase inactivating mutation in FtsE; and pEX F152E and pEX Δ141 to 171 indicate periplasmic domain variants shown to be unable to bind EnvC. Red stars indicate strains with a cell division defect. (*B*) Bacterial two-hybrid experiments, demonstrating the integrity of the F152E and Δ141 to 171 FtsX variants—both variants retain interactions with FtsE, FtsA, and FtsX confirming expression and complex assembly.

Next, we examined FtsEX’s role in daughter cell separation which is expected to require both its ATPase activity and interaction with EnvC (13, 19). For this experiment, we focused on the F152E and X-lobe deletion variants of FtsEX as these are the most obviously impaired in EnvC binding. Scanning confocal microscopy images are shown in Fig. 3*A*. Wild-type *E. coli* MR2 cells are short and rod shaped while cells lacking *ftsEX* (*E. coli* MR10) form long filaments or “chains” (19). Normal morphology was restored to MR10 cells by expressing wild-type FtsEX from a plasmid, but not by an FtsEX variant predicted to lack ATPase activity. Using the same complementation system, we found that both the F152E and Δ145-171 variants fail to rescue the chaining phenotype, suggesting they break the interaction with EnvC in vivo. Inability of F152E and Δ145-171 variants to support daughter cell separation is not due to an absence of expression since both variants support growth on low-salt media. However, to further characterize these variants, we performed a series of bacterial two-hybrid experiments testing dimerization (interaction with FtsX), complex formation (FtsE binding), and Z-ring interaction (FtsA binding) (Fig. 3*B*). Both the F152E and Δ145-171 FtsX variants retain all three functions unambiguously establishing their integrity. We therefore conclude that the interaction between FtsEX and EnvC is essential for daughter cell separation and that the F152E and Δ145-171 variants are well suited for probing the physiological effects of breaking the interaction between FtsEX and EnvC.

### FtsEX Has a Role in Intrinsic Resistance to Vancomycin and Bacitracin That Depends on both Its ATPase Activity and Interaction with EnvC.

*E. coli* and other gram-negative bacteria are intrinsically resistant to antibiotics such as vancomycin and bacitracin due to the presence of the outer membrane and the activity of efflux pumps that shield the peptidoglycan precursors from attack (30). However, in cases where this barrier is impaired, or when efflux is disrupted, such compounds are typically effective antimicrobial agents. FtsEX has a role in regulating several peptidoglycan remodelling enzymes that have already been linked to membrane integrity (21–25)—thus we considered the possibility that *ftsEX* deletion strains may also have cell envelope defects that would render them sensitive to such antibiotics.

**Fig. 4.**
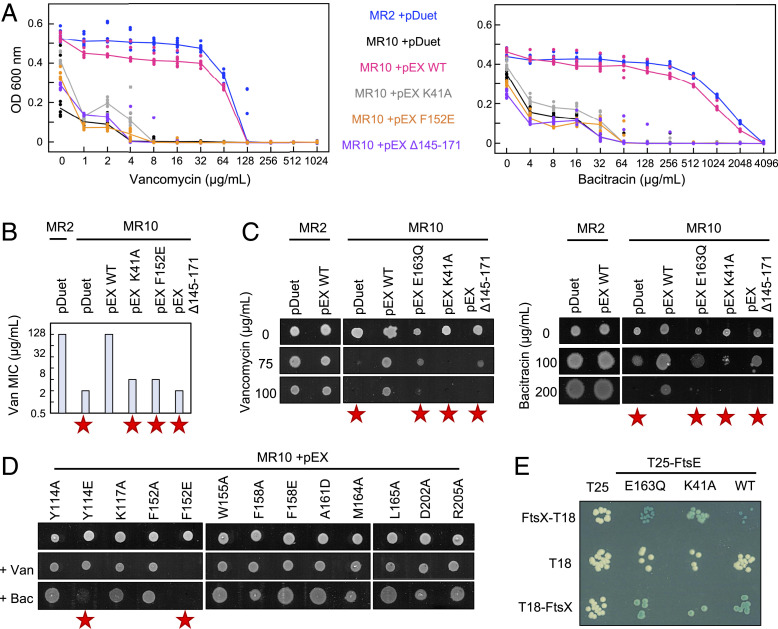
Intrinsic resistance to vancomycin and bacitracin depends on FtsEX and requires both its ATPase activity and interaction with periplasmic amidases. (*A*) Vancomycin and bacitracin MIC determinations for wild type (blue), *ftsEX*-deficient (black), and complemented (pink, gray, yellow, and purple) strains of *E. coli*. Lines indicate the median of eight repeats. (*B*) Vancomycin and bacitracin MIC values. Red stars indicate strains with impaired antibiotic resistance. (*C*) Growth of *E. coli* MR2 and MR10 carrying FtsEX variants spotted on antibiotic-supplemented agar and grown for 18 h. pDuet indicates an empty plasmid and pEX WT indicates wild-type *ftsEX*. pEX E163Q and pEX K41A have substitutions in FtsE predicted to impair ATPase activity. pEX Δ145-171 indicates FtsEX lacking a portion of the periplasmic domain (strictly substituted by a pair of glycines). (*D*) *E. coli* MR10 complemented with plasmid-borne *ftsEX* (with indicated substitution in FtsX periplasmic domain) spotted onto LB agar containing no antibiotic, 100 µg/mL vancomycin, or 200 µg/mL bacitracin, as indicated. (*E*) Bacterial two-hybrid experiments demonstrating integrity of FtsE variants—both K41A and E163Q variants interact with FtsX.

Minimum inhibitory concentration (MIC) determinations for vancomycin and bacitracin are shown in Fig. 4 *A* and *B* with further MICs reported in *SI Appendix*, Table S2. Wild-type *E. coli* is intrinsically resistant to both vancomycin and bacitracin, but strains lacking *ftsEX* are between 32- and 64-fold more susceptible. Complementing the *ftsEX*-deficient strain with plasmid-borne *ftsEX* restores intrinsic resistance, but variants predicted to be impaired in ATP binding (K41A) or hydrolysis (E163Q) do not. Failure to complement for the K41A and E163Q variants (both located in the FtsE ABC domain) is not due to poor expression or misfolding since they retain a positive interaction with FtsX—as shown by a bacterial two-hybrid analysis (Fig. 4*E*). These experiments show that FtsEX activity is required to maintain resistance to vancomycin and bacitracin, suggesting that FtsEX deficiency causes an outer membrane defect.

To assess the importance of EnvC-binding site in maintaining intrinsic resistance, we tested the 13 FtsEX periplasmic domain variants and X-lobe deletion for their ability to complement an *ftsEX*-deficient strain for growth on solid agar containing either vancomycin or bacitracin (Fig. 4 *C* and *D*). Both Δ145-171 and F152E variants were identified as having outer membrane defects. Follow-up experiments using MIC determinations in broth confirmed the need for an intact EnvC-binding site since both the Δ145-171 and F152E variants exhibit sensitivity on par with the inactive ATPase variants or empty vector control (*SI Appendix*, Table S2 and Fig. 4 *A* and *B*). Our results demonstrate that both the ATPase activity of FtsEX and its interaction with EnvC are required for intrinsic resistance to vancomycin and bacitracin.

### ATPase Activity and EnvC-Binding Functions of FtsEX Are Essential for Detergent Resistance.

**Fig. 5.**
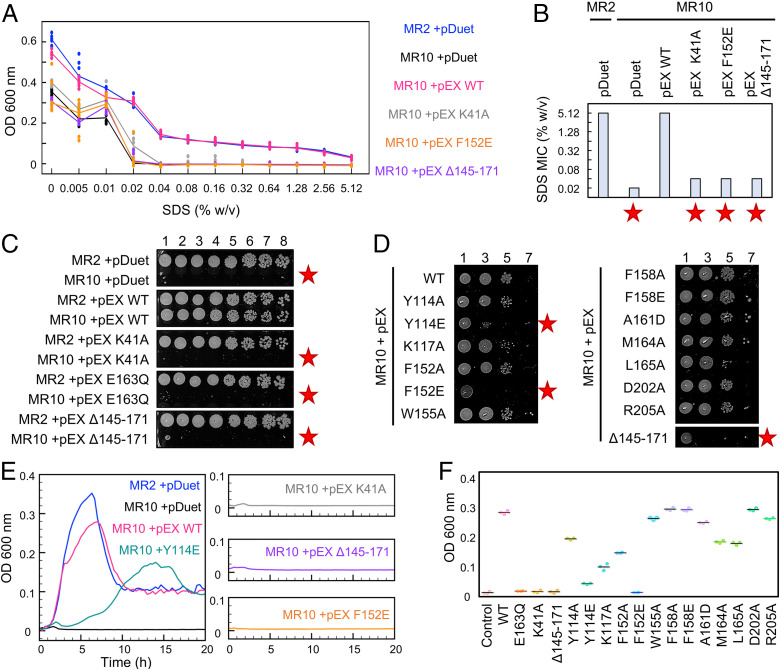
EnvC binding and ATPase activity of FtsEX are required for detergent resistance. (*A*) MIC determination for wild type (MR2), knockout (MR10), and plasmid-complemented *ftsEX* strains using SDS. Solid lines indicate median of eight replicates. (*B*) Detergent MIC values for indicated strains. Red stars indicate impaired detergent resistance. (*C*) *E. coli* viability on solid agar containing 0.1% SDS. Cultures were adjusted to OD_600_ = 1 before plating in series dilution. The number of 10-fold dilutions is indicated above the *Topmost* strip. (*D*) FtsEX-deficient *E. coli* (MR10) complemented by indicated FtsEX variants. (*E*) Growth curves for key strains in broth supplemented with 0.1% SDS. (*F*) Turbidity of *E. coli* cultures after 6 h growth in media containing 0.1% SDS.

To further characterize the outer membrane defect, we investigated whether strains lacking *ftsEX* are vulnerable to membrane-attacking detergents such as sodium dodecyl sulfate (SDS). MIC determinations for SDS in broth are shown in Fig. 5 *A* and *B*. We found a 256-fold difference between the MICs of wild-type (5.12% SDS) and *ftsEX*-deficient strains (0.02% SDS). Just as for daughter cell separation and antibiotic resistance phenotypes, detergent resistance can be restored to *ftsEX*-deficient strains by plasmid-borne *ftsEX*, but not by variants lacking ATPase activity (K41A) or that are unable to bind EnvC (Δ145-171 and F152E). Side-by-side comparison of each FtsEX variant’s viability on 0.1% SDS-agar confirms these results and additionally flags Y114E as a mildly disrupted interface variant (Fig. 5 *C* and *D*). Examining the growth curves for each variant in 0.1% SDS broth, we find that strains carrying wild-type *ftsEX* grow rapidly until stationary phase and then undergo lysis, while K41A, Δ145-171, and F152E variants show virtually no growth from the start (Fig. 5*E*). Variants with modestly impaired EnvC-binding characteristics, such as Y114E, present slower-than-WT growth over the first 6 h but remain viable until stationary phase, as per the wild type (Fig. 5 *E* and *F*). Identification of detergent resistance-breaking mutations in both the X-lobe and the central β-sheet of the FtsX Porter domain corroborate the EnvC-binding site observed in the FtsX-EnvC crystal structure.

### An Autoinhibitory Helix within EnvC Regulates Binding and Activation of Periplasmic Amidases.

**Fig. 6.**
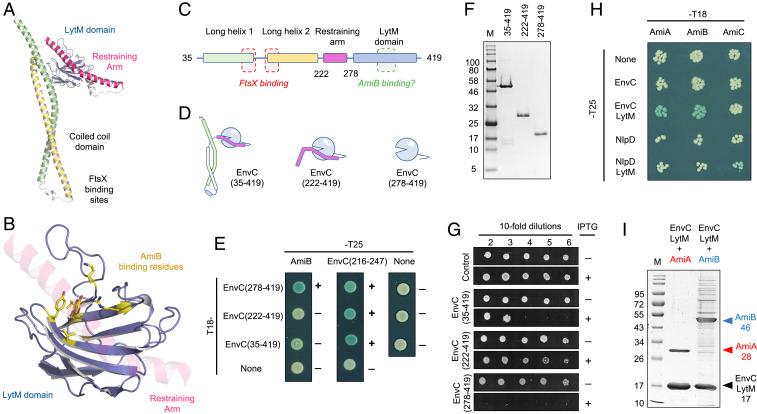
Evidence for an autoinhibitory element in EnvC. (*A*) Structure of EnvC. (*B*) Close-up of the LytM domain (blue) with putative AmiB-binding residues (18) shown as sticks (yellow). The restraining helix, composed of EnvC residues 222 to 278 (pink), is locked inside the proposed AmiB-binding groove. (*C*) Linear domain arrangement for mature EnvC. (*D*) Cartoon representations for three EnvC constructs used to probe AmiB binding and amidase activation. (*E*) Bacterial two-hybrid experiments showing interaction between EnvC variants and either AmiB or a fragment representing part of the restraining arm. (*F*) SDS gel showing purified EnvC constructs. (*G*) In vivo activation of amidases by EnvC constructs expressed in the bacterial periplasm. (*H*) Bacterial two-hybrid assay probing interactions between the three *E. coli* amidases (AmiA, AmiB, and AmiC) and two murein hydrolase activators (EnvC or NlpD) as either full-length proteins or truncated LytM domains. (*I*) Copurification of AmiA (lane 1) or AmiB (lane 2) with the EnvC LytM domain.

Having structurally characterized the interaction between FtsEX and EnvC, and probed the interface by mutagenesis, we next considered how FtsEX-EnvC might interact with downstream amidases involved in peptidoglycan remodelling. Peptidoglycan amidases (such as AmiA and AmiB) interact with EnvC via a large groove in its LytM domain and several EnvC variants have been identified that prevent amidase activation without impeding EnvC’s proper localization to the site of division (18). We mapped the position of these variants to the full-length structure of EnvC and found that the groove containing these residues is occluded by a long helix that wraps around the LytM domain (Fig. 6 *A*–*C* and Movie S3). We have named this structural feature the restraining arm.

We hypothesized that the EnvC restraining arm has an autoinhibitory role that prevents binding and activation of amidases (AmiA and AmiB) in the absence of stimulation by FtsEX. Using the structure as a guide, we made three different EnvC protein constructs: the first being equivalent to the mature EnvC protein (residues 35 to 419); the second lacking the coiled coil domain, but retaining the restraining arm (residues 222 to 419); and the third comprising only the EnvC LytM domain (residues 278 to 419) (Fig. 6*D*). We then performed bacterial two-hybrid experiments to assess which of these constructs interact with AmiB (Fig. 6*E*). We find that neither the full-length EnvC construct or the construct lacking the coiled coil domain interact with AmiB, but that a strong interaction is observed for AmiB and the isolated LytM domain (EnvC 278 to 419). The absence of an AmiB interaction for either EnvC or the EnvC 222 to 419 variant is not due to lack of expression or other artifact since all three EnvC constructs give positive interactions with a short protein fragment encompassing the isolated restraining arm (Fig. 6*E*, *Center* column). We were also able to purify all three EnvC variants as His-tagged proteins, further confirming their stability (Fig. 6*F*). We therefore conclude that the restraining arm does indeed represent an autoinhibitory element that binds to the LytM domain and prevents binding of AmiB.

We next assessed the effect of periplasmic expression of each of our three EnvC constructs on bacterial viability. Cells carrying appropriate expression plasmids were plated-in-dilution on solid agar in which an inducing agent (IPTG, isopropyl-β-D-thiogalactoside) was either present or absent (Fig. 6*G*). Cells carrying an empty plasmid are viable under both conditions, but those baring the isolated LytM domain are only viable in the absence of the inducer, presumably because the LytM domain can both bind and activate amidases causing peptidoglycan degradation. In contrast, expression of the “wild-type” EnvC 35 to 419 construct gave only modest impairment in plating efficiency, and bacteria expressing the 222 to 419 construct are barely impaired at all. Both of these constructs retain the restraining arm. The in vivo expression studies clearly show that amidase activity is stimulated by the free LytM domain (18), but autoinhibited by the presence of the restraining arm identified here. These results correlate strongly with the bacterial two-hybrid data showing an interaction between the amidase and the EnvC LytM domain but not with variants that retain the restraining arm. Most interestingly, however, we find that it is the construct lacking the coiled coil domain that has the weakest effect on bacterial viability, rather than the wild-type construct which retains both the restraining arm and coiled coil domain. Our interpretation of this result is that both constructs are autoinhibited by the restraining arm, but that only the wild-type construct can be activated by FtsEX. The data support a model where the EnvC restraining arm acts as an autoinhibitory element that must be displaced before the LytM domain can bind and activate cognate amidases.

### Autoinhibition of the LytM Domain Is a General Feature of Peptidoglycan Amidase Regulation.

In light of the role for the restraining arm in regulating binding of AmiB to the EnvC LytM domain, we considered whether this mechanism might be applied to other peptidoglycan amidases and their activators. Using a bacterial two-hybrid screen, we systematically tested three *E. coli* peptidoglycan amidases (AmiA, AmiB, and AmiC) for interaction with the murein hydrolase activators (EnvC and NlpD), or their respective LytM domains (EnvC-LytM and NlpD-LytM). Our prediction was that each amidase should bind its cognate activator’s LytM domain, but not the full-length activators, as they retain autoinhibitory elements. We found that both AmiA and AmiB bind to the EnvC LytM domain, and AmiC binds to the NlpD LytM domain, but none of the amidases interact with full-length EnvC or NlpD (Fig. 6*H*). Interactions between the EnvC LytM domain and its cognate amidases were further confirmed by copurifying noncovalent complexes of AmiA and AmiB with the EnvC LytM domain (Fig. 6*I*). In these Ni-based immobilized metal affinity chromatography (Ni-IMAC) purifications, only the EnvC LytM domain is His-tagged, yet both AmiA and AmiB constructs copurify as stable complexes. Our data are consistent with the previous elucidation of cognate activator/amidase pairings in vitro (17) and show that the system of autoinhibition we ascribe to the EnvC-AmiB interaction applies equally to EnvC-AmiA and possibly NlpD-AmiC, too.

### A Model for FtsEX-EnvC Suggests Conformational Changes at the Heart of Amidase Regulation.

**Fig. 7.**
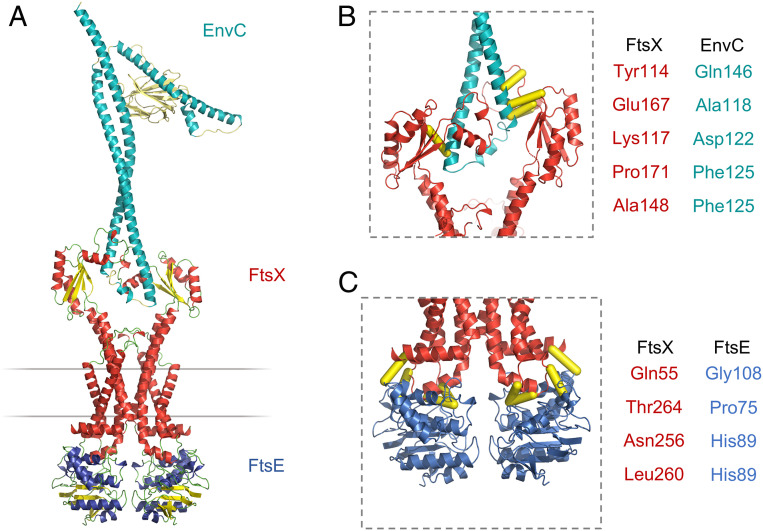
Proposed structure of the FtsEX-EnvC complex. (*A*) Homology model for FtsEX-EnvC based on the crystal structure of EnvC bound to the FtsX periplasmic domain presented here and the MacB ABC transporter from *Acinetobacter* *baumannii* (8). (*B*) High-scoring predicted intermolecular contacts between FtsX and EnvC based on coevolutionary analysis using Gremlin (32). Predicted intermolecular contacts are shown as yellow tubes. Residue identities are shown to the *Right*. (*C*) Similar contacts predicted for interaction of FtsE and FtsX.

To better understand the location and orientation of EnvC when bound to FtsX, we constructed a molecular model of FtsEX-EnvC based on the crystallographic data presented here and preexisting structures of the homologous ABC transporter, MacB (Fig. 7). Models of *E. coli* FtsEX were constructed using Phyre (31) and the position of bound EnvC determined by superposing the FtsX-EnvC complex onto its periplasmic domains (Fig. 7*A*). Interactions between FtsE, FtsX, and EnvC were then validated by mapping putative contact sites implied by coevolution analysis using Gremlin (32) (Fig. 7 *B* and *C*). Molecular dynamics (MD) simulations of the FtsX-EnvC complex and an FtsEX-EnvC model, embedded in a lipid bilayer, further support the proposed model, with both secondary structure and protein–protein interactions maintained throughout the simulations. In addition, the dynamics of the EnvC correlate well to the B-factors observed in the crystal structure (*SI Appendix*, Fig. S4 and Movie S4).

The FtsEX-EnvC model presented here most likely represents an ADP-bound or nucleotide-free form of FtsEX in which EnvC is locked in an inactive resting state (Movie S5). The model makes three useful predictions relating to function. Firstly, the location of EnvC places the LytM domain a considerable distance from the cytoplasmic membrane, close to the expected location of the peptidoglycan layer. Secondly, the model predicts that interaction with EnvC occurs solely via the FtsX periplasmic domain located between transmembrane segment 1 and 2 and does not require the extracytoplasmic loop between transmembrane helices 3 and 4. Finally, the model suggests a continuous route by which ATP-driven conformational changes in FtsEX can be propagated across the membrane and through the coiled coil domain of EnvC (see *Discussion*). Such mechanotransmission-driven conformational changes are likely essential to release the EnvC LytM domain from the restraining arm and to enable binding and activation of amidases.

## Discussion

FtsEX functions in bacterial cell division and regulates periplasmic amidases such as AmiA and AmiB via its interaction with the murein hydrolase activator, EnvC. Here we present a 2.1 Å crystal structure of EnvC bound to the periplasmic domains of FtsX, revealing the molecular underpinnings of this interaction and the first complete structure of EnvC (Fig. 1). The structure defines an unexpected 2-to-1 binding stoichiometry for the FtsX-EnvC complex and implicates both the X-lobe and Porter domain in EnvC binding. Mutational analysis of FtsX-EnvC interface combined with a bacterial two-hybrid screen experimentally confirms the EnvC-binding site and identifies key interface residues (Fig. 2). Binding-impaired FtsEX variants are unable to separate daughter cells following division (Fig. 3), but support viability on low-osmolarity media. FtsEX also has a role in maintaining outer membrane integrity that requires its interaction with EnvC. Loss of FtsEX by deletion, inactivation of its ATPase activity, or impairment of its EnvC-binding site, all increase bacterial susceptibility to antibiotics (Fig. 4) and detergents (Fig. 5) that would not usually pass the outer membrane barrier. The structure of EnvC further reveals a previously unrecognized autoinhibitory element, here dubbed the restraining arm, that modulates EnvC’s interaction with downstream amidases, such as AmiA and AmiB, to regulate their activation (Fig. 6). In its resting state, EnvC cannot bind and activate its cognate amidases because the restraining arm sits within the amidase binding groove. A model of FtsEX-EnvC constructed on the basis of similarity to the MacB ABC transporter makes a compelling case that FtsEX mechanotransmission drives conformational changes in EnvC that displace the restraining arm, leading to amidase activation (Fig. 7).

**Fig. 8.**
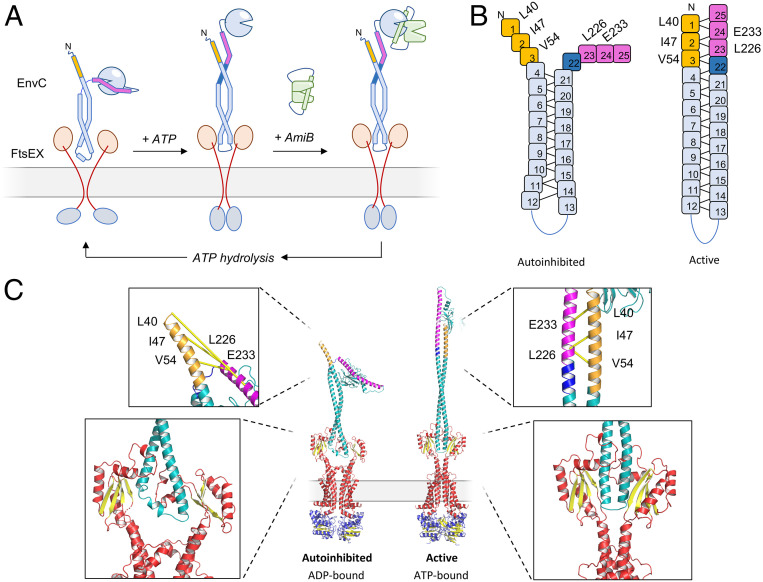
Mechanism for FtsEX-EnvC-mediated amidase activation. (*A*) Overview of proposed conformational changes in FtsEX-EnvC during the ATP binding and hydrolysis cycle. See discussion for details. (*B*) Schematic representations of the EnvC N-terminal domain and restraining arm in the autoinhibited and active forms. Heptad repeats are shown as numbered boxes; coevolving residues identified using Gremlin are annotated beside their respective heptad. (*C*) Hypothetical models for the autoinhibited and active forms of the FtsEX-EnvC complex. Boxed regions show close-up views of the proposed interaction between the heptads 1 to 3 and 22 to 25 and the hypothesized compression of the EnvC coiled coil due to FtsEX mechanotransmission. Coevolving residue pairs in the N-terminus and restraining arm are shown linked by yellow lines. Heptads 1 to 3 are shown in gold, heptads 23 to 25 in pink, and heptad 22 in dark blue.

The data support a revised mechanism for amidase control by FtsEX-EnvC that acknowledges the role of the EnvC restraining arm in autoinhibition and the propagation of conformational change via FtsX-EnvC (Fig. 8). Our mechanism builds on previous work from the laboratories of Lutkenhaus (13), Bernhardt (12, 17, 18, 33), Alber (11), Winkler (15, 16), Hermoso (34), and others. In the ADP-bound form, FtsEX-EnvC adopts a resting state in which the EnvC LytM domain is tightly bound to the restraining arm, preventing its interaction with periplasmic amidases. Binding of ATP to FtsE in the cytoplasm induces mechanotransmission by FtsEX, leading to a conformational change that is transmitted across the membrane, through the EnvC coiled coil, and on toward the LytM domain and restraining arm. This conformational change causes release of the LytM domain from the restraining arm, allowing it to bind and activate amidases such as AmiB—most likely by displacing the amidase’s own blocking helix (33) to reveal the site of peptidoglycan hydrolysis. ATP hydrolysis eventually leads the system to reset, returning the LytM domain to the restraining the arm and releasing the amidase.

Exactly how movement of the FtsX periplasmic domains causes release of the restraining arm from the LytM domain will require further investigation. However, we speculate that the restraining arm (and the preceding linker) might be induced to form an extension of the existing coiled coil by packing against the exposed EnvC N-terminus (Fig. 8 *A* and *B*). This would free the LytM domain for amidase binding while offsetting the energetic cost of pulling the restraining arm from the amidase-binding groove. Supporting this hypothesis, we identify three unpaired heptad repeats within the EnvC N-terminus (heptads 1 to 3) and another four within the restraining arm (heptads 22 to 25) that could form an extended coiled coil (Fig. 8*B*). We also find coevolving residue pairs that imply an intramolecular contact between the EnvC N-terminus and the restraining arm (Fig. 8*B*). Coevolution of these residues is difficult to understand in context of the conformation observed here, but makes good sense if heptads 1 to 3 were to be reunited with heptads 22 to 25 by a conformational change induced by FtsEX (Fig. 8 *A* and *B*).

A hypothetical model showing precisely how a conformational change might be propagated through FtsEX-EnvC is shown in Movie S6. Three-dimensional (3D) models for the proposed ADP-bound autoinhibited state and the active ATP-bound state are also shown in Fig. 8*C*. The key assumptions underpinning the model are that FtsX and EnvC remain bound throughout the ATP hydrolysis cycle, and that the periplasmic domains of the FtsEX complex are squeezed together by a mechanotransmission mechanism similar to that of MacB (1). Our mechanism contends that as the FtsX periplasmic domains compress, EnvC is forced into a geometrically ideal coiled coil that favors interaction between the restraining arm and the EnvC N-terminus—thereby reuniting heptads 1 to 3 with heptads 22 to 25. This conformational change frees the LytM domain from the restraining arm, repositioning it deep within the periplasm where it is needed to activate amidases in proximity to the peptidoglycan layer (Fig. 8 *A* and *C*).

We predict a similar mechanism in gram-positive organisms and mycobacteria where the peptidoglycan hydrolase interacts directly with FtsEX. This includes the FtsEX-PcsB system of *S. pneumoniae* (34), FtsEX-CwlO of *Bacillus subtilis* (35), and the FtsEX-RipC systems of *Mycobacterium smegmatis* (11) and *Corynebacterium glutamicum* (36). In these systems, we predict mechanotransmission by FtsEX drives direct release of the enzymatic domain from an internal autoinhibitory element giving access to peptidoglycan-like substrates. EnvC, PcsB, CwlO, and RipC all have similar modular domain structures to EnvC and interact with a cognate FtsEX complex. RipC and CwlO have also been shown to be autoinhibited by their N-termini (11, 35). In the case of PcsB, the available structure (34) enables us to predict the probable FtsEX-binding site within the coiled coil. Specifically, we predict that residues 101 to 114 and 130 to 148 of PcsB form an equivalent pair of FtsX-binding sites to those of EnvC (114 to 127 and 142 to 160). NMR experiments implicate the X-lobe of *S. pneumoniae* FtsX in PcsB binding (27) consistent with the interface observed here for FtsX-EnvC. PcsB also has an alanine-rich helix (210 to 253) located between its coiled coil (42 to 208) and the C-terminal CHAP domain (279 to 392) that is homologous to the restraining arm of EnvC. Should PcsB engage FtsEX as a monomer, as EnvC does, then the alanine-rich helix of PcsB could perform an equivalent function to the restraining arm in gating access to the C-terminal domain.

The FtsEX-EnvC-AmiA/B system is important for cell division in *E. coli* but also linked to cell envelope integrity (21–25). FtsEX-deficient *E. coli* are 32- and 64-fold more susceptible to vancomycin and bacitracin, and 256-fold more susceptible to SDS. Both the ATPase activity of FtsEX and its interaction with EnvC via the X-lobe are required for resistance. These data suggest inhibition of FtsEX as a useful strategy for breaking the intrinsic resistance of gram-negative bacteria allowing treatment with antibiotics that would usually only be effective against gram-positives. Alternatively, molecules interfering with the EnvC autoinhibition mechanism might cause bacterial lysis via uncontrolled activation of peptidoglycan hydrolases (18). Our structure precisely defines the amidase-binding site of EnvC and the FtsEX-EnvC interface for future inhibitor development.

FtsEX-linked hydrolases are widespread among bacteria and have functions outside of cell division. The FtsEX-CwlO system of *B. subtilis* is involved in cell elongation and sporulation (35, 37), and the FtsEX-RipC system has been linked to arabinoglycan biogenesis in *C. glutamicum* (36). Both of these systems use additional protein factors that collaborate with FtsEX to confer a further layer of regulatory complexity (36, 38). While FtsEX-based systems have many functions, the core interaction between FtsEX and its coiled coil partners nonetheless seems to be conserved, and the structure presented here therefore informs on several FtsEX-based systems that activate coiled coil partners through interactions with the FtsEX extracytoplasmic domain.

The FtsX-EnvC interaction is the third structurally characterized example of a periplasmic partner bound to the extracytoplasmic domain of a type VII ABC transporter. The other two examples are the MacB-MacA interaction observed within the MacAB-TolC tripartite efflux pump (7) and the LolC-LolA interaction from the LolCDE-LolA lipoprotein trafficking complex (4). All three interactions involve different molecular interfaces and contrasting stoichiometries (*SI Appendix*, Fig. S5). The MacB dimer engages with the MacA hexamer (2:6 stoichiometry) (7) via an extensive contact surface on the exterior of its Porter and Sabre domains, while LolC (but not LolE) interacts with LolA via the bespoke “hook and pad” located within its Sabre domain (1:1) (4). As shown here, the FtsEX-EnvC interaction involves a single EnvC monomer locked between the two periplasmic domains via interactions with both the X-lobe and the Porter domain (2:1). The FtsEX-EnvC interaction is only possible because of the absence of the Sabre domain (relative to MacB and LolC family transporters) and the presence of the X-lobe—which is unique to FtsEX. Taken together, these structures define an astonishingly diverse set of binding modes for type VII ABC transporters that are intimately linked to the fine structure of their periplasmic domains.

In summary, the structure of EnvC bound to the FtsX periplasmic domains provides valuable structural insight into the bacterial FtsEX-EnvC complex and its roles in peptidoglycan hydrolase activation.

## Methods

Full methods are available in *SI Appendix*. Copurification of the histidine-tagged FtsX periplasmic domain and nontagged EnvC used Ni-IMAC after coexpression in *E. coli* C43(DE3) from pETDuet1-based vector. The structure of EnvC in complex with the FtsX periplasmic domain was determined by X-ray crystallography using tools from the CCP4 software suite (39–44). Phasing used a molecular replacement strategy with the EnvC LytM domain (18) and *M. tuberculosis* FtsX periplasmic domain (11) as search probes. Bacterial two-hybrid experiments used the bacterial adenylate cyclase two hybrid (BACTH) system (45), with *E. coli* BTH101 cells grown on IPTG/X-gal LB plates at room temperature. In vivo complementation experiments used *E. coli* strains MR2 (MG1655 *ΔLacI*) and MR10 (MG1655 *ΔLacI ΔFtsEX::Kan*^*R*^) with wild type or variant *ftsEX* genes in pETDuet1. Growth in low-salt broth was monitored in LBON50 (LB with no salt, diluted twofold with water) at 37 °C. Minimum inhibitory concentrations were determined in LB supplemented with 50 μg/mL ampicillin and 1 mM IPTG at 37 °C. Modeling of FtsEX based on structures of MacB used Phyre (31) and coevolution analysis used Gremlin (32). Structural figures were produced with Pymol (46).

## Supplementary Material

Supplementary File

Supplementary File

Supplementary File

Supplementary File

Supplementary File

Supplementary File

Supplementary File

## Data Availability

Structural data have been deposited in the Protein Data Bank, http://www.wwpdb.org (PDB ID code 6TPI).
