# Supplementary material for: Insights into bacterial cell division from a structure of EnvC bound to the FtsX periplasmic domain
Source: Proc Natl Acad Sci U S A. 2020 Oct 23;117(45):28355–65. doi: 10.1073/pnas.2017134117 (PMC7668044; doi:10.1073/pnas.2017134117)
Supplement: Supplementary File [file pnas.2017134117.sapp.pdf]

Supplemental Information

Insights into bacterial cell division from a structure of EnvC bound to the FtsX periplasmic domain

Jonathan Cook, Tyler C. Baverstock, Martin McAndrew, Phillip J. Stansfeld, David I. Roper, Allister Crow

Supplemental Tables

Table S1 X-ray data and structure refinement statistics.

Table S2 Minimum Inhibitory Concentrations (MICs) for antimicrobial compounds.

Supplemental Figures

Figure S1 Identification of heptad repeats in the EnvC N-terminus.

Figure S2 Comparison of FtsX periplasmic domains.

Figure S3 Viability and growth of FtsEX variants on low-osmolarity media.

Figure S4 Molecular simulations of the FtsX-EnvC and FtsEX-EnvC complexes.

Figure S5 Type VII ABC transporters have diverse interactions with their periplasmic partners.

Supplemental Text

Figure legend for Movie 1

Figure legend for Movie 2

Figure legend for Movie 3

Figure legend for Movie 4

Figure legend for Movie 5

Figure legend for Movie 6

Supplemental Methods

www.pnas.org/cgi/doi/10.1073/pnas.2017134117

Table S1: X-ray data and structure refinement statistics.

	6TPI
**Data collection**	
Beam line	Diamond I04-1
Wavelength (Å)	0.91587
**Crystal parameters**	
Space group	$P2_12_12_1$
Unit cell dimensions (Å)	67.03, 100.47, 107.78
Unit cell angles (°)	90, 90, 90
Mosaic spread (°)	0.43
**Reflection data***	
Resolution range (Å)	55.76-2.10 (2.16-2.10)
Unique reflections	43,252 (3,494)
R_{sym}	0.103 (1.242)
R_{meas}	0.112 (1.372)
R_{pim}	0.044 (0.577)
I/σ(I)	15.0 (2.2)
$CC_{½}$	0.999 (0.767)
Completeness (%)	100.0 (100.0)
Multiplicity	12.0 (10.8)
Wilson B (Å^2)	33.6
**Refinement†**	
Resolution (Å)	55.82 – 2.10
Number of reflections	41,086
$R_{overall}$	0.215
R_{free}	0.275
Rms (bond lengths) (Å)	0.007
Rms (bond angles) (°)	1.48
**Model B-factors**	
EnvC (chain A) (Å^2)	54
FtsX peri (chain B) (Å^2)	51
FtsX peri (chain C) (Å^2)	119
Waters (Å^2)	47
**Ramachandran statistics‡**	
Favoured (%)	97.4
Allowed (%)	2.4
Outlier (%)	0.2

Values in parentheses indicate the highest resolution bin.
Refinement statistics are from Refmac (43).
Ramachandran statistics as reported by Rampage (48).

Table S2: Minimum Inhibitory Concentrations (MICs) for antimicrobial compounds.

Antibiotic	*E. coli* strain	Plasmid *ftsEX* variant	Median MIC (µg/mL)
Vancomycin	MR2	-	256
	MR10	-	4
	MR10	*ftsEX*	256
	MR10	*ftsEX* (K41A)	8
	MR10	*ftsEX* (Δ145-171)	4
	MR10	*ftsEX* (F152E)	8
Bacitracin	MR2	-	4096
	MR10	-	64
	MR10	*ftsEX*	4096
	MR10	*ftsEX* (K41A)	64
	MR10	*ftsEX* (Δ145-171)	64
	MR10	*ftsEX* (F152E)	64

Detergent	Strain	Plasmid *ftsEX* variant	Median MIC (% w/v)
SDS	MR2	-	>5.12
	MR10	-	0.02
	MR10	*ftsEX*	>5.12
	MR10	*ftsEX* (K41A)	0.04
	MR10	*ftsEX* (Δ145-171)	0.04
	MR10	*ftsEX* (F152E)	0.02

Median MIC values are from eight determinations. MR2 is *E. coli* MG1655 *ΔlacI*. MR10 is *E. coli* MG1655 *ΔlacI ΔftsEX::Kan*^{R}.

Table S3: Plasmids used in this study.

Plasmid	Internal name	Backbone	Contents	Notes	Figures
FtsEX expression and complementation					
pEX WT	pJC6 065	pETDuet1	FtsEX	'Wild type' FtsEX. N-terminal His-tag on FtsE. The FtsEX genes are cloned across both cloning sites rather than into separate cloning sites. See Methods.	3a, 4a, 4b, 4c, 5a, 5b, 5c, 5e , 5f, Table S2, S3a, S3b, S3c
pDuet	pETDuet1	pETDuet1	-	Used as an empty vector control.	3a, 4a, 4b, 4c, 5a, 5b, 5c, 5e, 5f, Table S2, S3a, S3b, S3c
pEX K41A	pJC6 138	pETDuet1	FtsE(K41A)X	FtsEX variant. Predicted to be impaired in ATP binding. N-terminal His-tag on FtsE.	3a, 4a, 4b, 4c, 5a, 5b, 5c, 5e, 5f , Table S2, S3a, S3c
pEX E163Q	pJC6 140	pETDuet1	FtsE(E163Q)X	FtsEX variant. Predicted to be impaired in ATP hydrolysis. N-terminal His-tag on FtsE.	4c, 5c, 5f, S3a, S3c
pEX Δ145-171	pJC6 232	pETDuet1	FtsEX(Δ145-171::GG)	FtsEX variant. X-lobe is substituted by a pair of glycines. Designed using the EnvC-FtsX periplasmic domain crystal structure. N-terminal His-tag on FtsE.	3a, 4a, 4b, 4c, 5a, 5b, 5c, 5e, 5f , Table S2, S3a, S3b, S3c
pEX Y114A	pMiTB1027	pETDuet1	FtsEX(Y114A)	FtsEX periplasmic domain variant. N-terminal His-tag on FtsE.	4d, 5d, 5f, S3b, S3c
pEX Y114E	pMiTB1028	pETDuet1	FtsEX(Y114E)	"	4d, 5d, 5f , S3b, S3c
pEX K117A	pMiTB1029	pETDuet1	FtsEX(K117A)	"	4d, 5d, 5f , S3b, S3c
pEX F152A	pMiTB1030	pETDuet1	FtsEX(F152A)	"	4d, 5f, S3b, S3c
pEX F152E	pMiTB1031	pETDuet1	FtsEX(F152E)	"	3a, 4a, 4b, 4d, 5a, 5b, 5d, 5e, 5f, Table S2, S3b, S3c

pEX W155A	pMiTB1032	pETDuet1	FtsEX(W155A)	"	4d, 5d, 5f, S3b, S3c
pEX F158A	pMiTB1033	pETDuet1	FtsEX(F158A)	"	4d, 5d, 5f , S3b, S3c
pEX F158E	pMiTB1034	pETDuet1	FtsEX(F158E)	"	4d, 5d, 5f , S3b, S3c
pEX A161D	pMiTB1035	pETDuet1	FtsEX(A161D)	"	4d, 5d, 5f , S3b, S3c
pEX M164A	pMiTB1036	pETDuet1	FtsEX(M164A)	"	4d, 5d, 5f , S3b, S3c
pEX L165A	pMiTB1037	pETDuet1	FtsEX(L165A)	"	4d, 5d, 5f , S3b, S3c
pEX D202A	pMiTB1038	pETDuet1	FtsEX(D202A)	"	4d, 5d, 5f , S3b, S3c
pEX R205A	pMiTB1039	pETDuet1	FtsEX(R205A)	"	4d, 5d, 5f , S3b, S3c
EnvC and FtsX periplasmic domain co-expression (Structure determination)					
	pJC6 120	pETDuet1	FtsX (110-209) in first site plus EnvC (35-419) in second site.	C-terminal His-tag on FtsX (110-209). Used for co-purification and structure of EnvC bound to the FtsX periplasmic domain (6TPI).	1a, 1b, 1c, 1d, 1e, 2a, 6a, 6b, S1, Table S1
Bacterial 2-Hybrid Experiments (EnvC and the FtsX periplasmic domain variants)					
	pJC6 124	pKNT25	EnvC (35-419)	-T25	2b,c
	pJC6 192	pUT18	FtsX (110-209)	-T18 ('Wild Type' FtsX periplasmic domain)	2b,c
	pJC6 194	pUT18	FtsX (110-209) Δ145-171:GG	-T18 (X-lobe deletion in FtsX periplasmic domain)	2b
	pMiTB1001	pUT18	FtsX (110-209) (Y114A)	-T18 (Interface variant in FtsX periplasmic domain)	2c
	pMiTB1002	pUT18	FtsX (110-209) (Y114E)	"	2c
	pMiTB1003	pUT18	FtsX (110-209) (K117A)	"	2c
	pMiTB1004	pUT18	FtsX (110-209) (F152A)	"	2c
	pMiTB1005	pUT18	FtsX (110-209) (F152E)	"	2c
	pMiTB1006	pUT18	FtsX (110-209) (W155A)	"	2c
	pMiTB1007	pUT18	FtsX (110-209) (F158A)	"	2c
	pMiTB1008	pUT18	FtsX (110-209) (F158E)	"	2c
	pMiTB1009	pUT18	FtsX (110-209) (A161D)	"	2c

pMiTB1010	pUT18	FtsX (110-209) (M164A)	“	2c
pMiTB1011	pUT18	FtsX (110-209) (L165A)	“	2c
pMiTB1012	pUT18	FtsX (110-209) (D202A)	“	2c
pMiTB1013	pUT18	FtsX (110-209) (R205A)	“	2c

Bacterial 2-hybrid EnvC variant interactions with AmiB

pJC6 128	pUT18C	EnvC (35-419)	T18-	6e
pJC6 274	pUT18C	EnvC (222-419)	T18-	6e
pJC6 253	pUT18C	EnvC (278-419)	T18-	6e
pJC6 249	pKNT25	EnvC (216-247)	-T25	6e
pJC6 243	pKNT25	AmiB (23-445)	-T25	6e

Bacterial 2-hybrid (AmiA, AmiB and AmiC vs EnvC, EnvC LytM, NlpD and NlpD LytM)

pTB1011	pUT18	AmiA	-T18	6h
pJC6238	pUT18	AmiB	-T18	6h
pTB1009	pUT18	AmiC	-T18	6h
pJC6124	pKNT25	EnvC (35-419)	-T25	6h
pJC6257	pKNT25	EnvC (278-419)	-T25 (EnvC LytM domain)	6h
pTB1005	pKNT25	NlpD (27-379)	-T25	6h
pTB1010	pKNT25	NlpD (250-379)	-T25 (NlpD LytM domain)	6h

Periplasmic Expression of EnvC variants

pET21a	pET21a	-	Empty vector control	6g
pJC6 260	pET22b	EnvC (35-419)	pelB sec signal	6g
pJC6 262	pET22b	EnvC (222-419)	pelB sec signal	6g
pJC6 263	pET22b	EnvC (278-419)	pelB sec signal	6g

His-tagged EnvC variants for expression as stable proteins

pJC6 477	pETDuet1	EnvC (35-419)	N-terminal His-tag, cytoplasmic expression	6f
pJC6 474	pETDuet1	EnvC (222-419)	N-terminal His-tag, cytoplasmic expression	6f
pJC6 276	pETDuet1	EnvC (278-419)	N-terminal His-tag, cytoplasmic expression	6f

Co-expression and co-purification of amidases (AmiA and AmiB) with the EnvC LytM domain

pTB1016	pETDuet1	EnvC (278-419) in first site, and AmiA (23-446) in the second	N-term His-tag on EnvC LytM domain and no tag on AmiA	6i
pJC6278	pETDuet1	EnvC (278-419) in first site, and	N-term His-tag on EnvC LytM domain and no tag on AmiB	6i

		AmiB (23-446) in the second		

Bacterial 2-hybrid demonstrating stability of key variants

pJC6 199	pUT18	FtsX	-T18	3b, 4e
pJC6 382	pUT18C	FtsX	T18-	4e
pJC6 351	pKT25	FtsE	T25-	3b, 4e
pJC6 369	pKNT25	FtsE (K41A)	-T25	4e
pJC6 372	pKT25	FtsE (E163Q)	T25-	4e
pJC6 295	pKNT25	FtsX	- T25	3b
pJC6 227	pKT25	FtsA	T25 -	3b
pJC6 297	pUT18	FtsX(Δ145-171::GG)	- T18	3b
pJC6 300	pUT18	FtsX(F152E)	- T18	3b

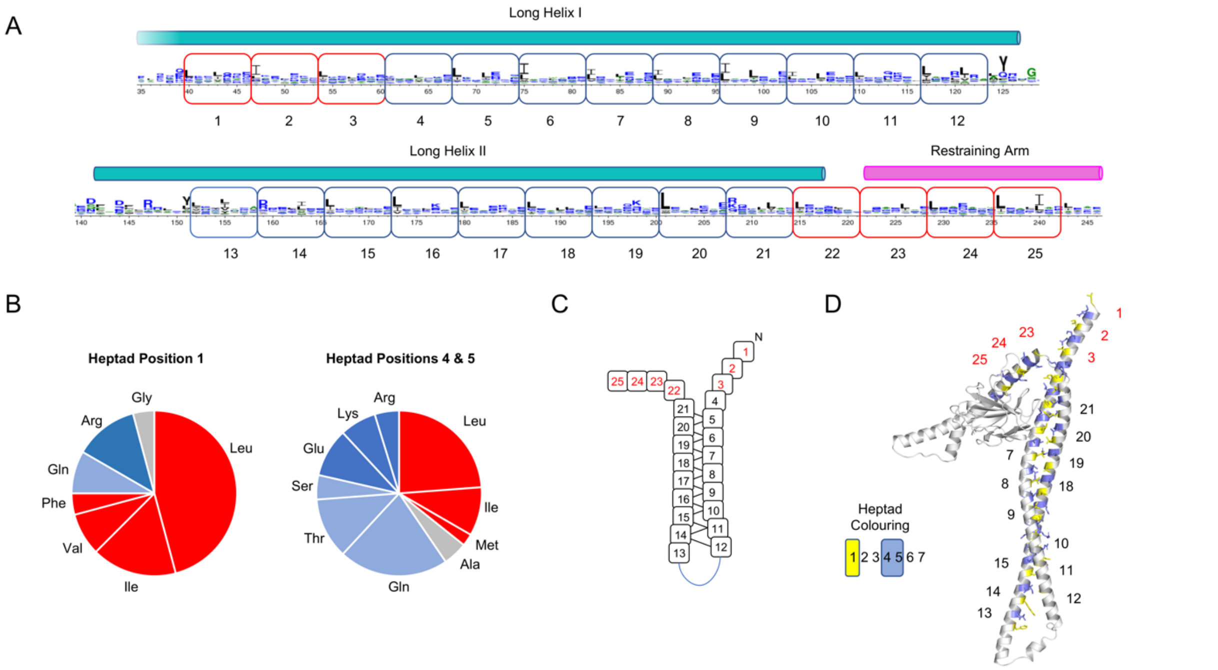

Figure S1: Identification of heptad repeats in the EnvC N-terminus. (**A**) Location of twenty-five 7-residue repeats are shown as rounded boxes. Residue numbering is that of *E. coli* EnvC, conservation at each position across known EnvC homologues is shown as a weblogo generated by Gremlin (33). Heptads that contribute to the coiled coil in the structure shown with *blue* boxes and those that remain unpaired are marked with *red* boxes. (**B**) Amino acid composition of the EnvC heptads. (**C**) Schematic representation of the EnvC N-terminus and restraining arm as a series of heptads. 'Paired' heptads that contribute to the coiled coil are shown in *black*, unpaired heptads that do not contribute to the coiled coil are shown in *red*. (**D**) Structure of EnvC (extracted from 6TPI) coloured to emphasise the location of heptad repeats and the knobs-into-holes interaction. Residue 1 of each heptad (typically forming a 'knob') is coloured yellow, and residues 4 and 5 (forming 'holes') are coloured blue. The unpaired heptads are highly unusual and likely to be mechanistically important.

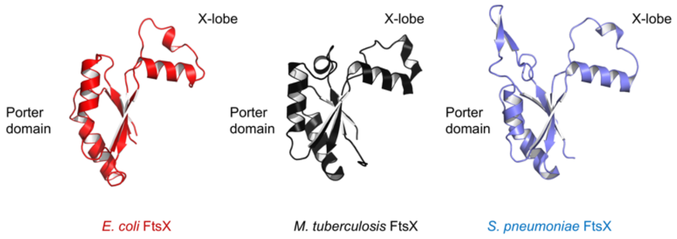

Figure S2: Comparison of FtsX periplasmic domains. Structures are derived from PDB entries 6TPI (*Escherichia coli* FtsX), 4N8N (*Mycobacterium tuberculosis* FtsX) and 6HEE (*Streptococcus pneumoniae* FtsX). The periplasmic domain of *M. tuberculosis* FtsX can be superposed with *E. coli* FtsX with an RmsD of 2.2 Å (for 88 matched amino acids) and *S. pneumoniae* FtsX can be superposed with an RmsD of 1.9 Å (for 85 matched amino acids).

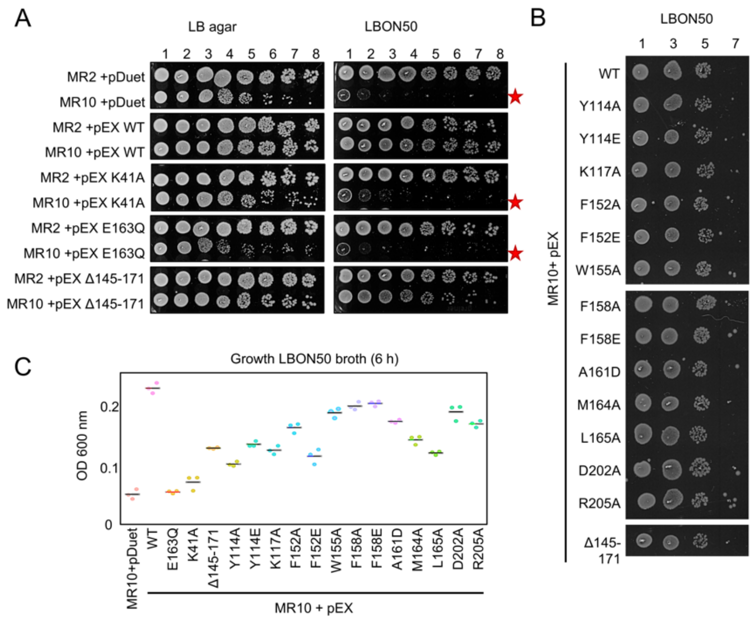

Figure S3: Viability and growth of FtsEX variants on low-osmolarity media. (**A**) Growth of *E. coli ftsEX* variants on LB agar and low osmolarity (LBON50) media. Wild type (MR2) or *ftsEX*-deficient (MR10) *E. coli* were complemented with indicated plasmids, grown to OD 1 then spotted out as a series of 10-fold dilutions. The number of dilutions are indicated above each image. pDuet indicates an empty vector control. pEX WT indicates wild type *ftsEX* on plasmid. pEX K41A and pEX E163Q encode FtsEX variants expected to lack ATPase activity due to substitutions in FtsE. All other variants encode mutations located within the FtsX periplasmic domain at the interface with EnvC. Stars highlight *ftsEX* variants with impaired plating efficiency. (**B**) Viability assays for a series of FtsEX periplasmic domain variants grown on low osmolarity media (LB0N50). All periplasmic domain variants were able to complement ftsEX deficieny and restore viability on low osmolarity media to wild type levels. (**C**) Growth of plasmid-complemented *E. coli* MR10 in low osmolarity broth (LBON50). pDuet indicates an empty vector, and all other strains carry the indicated *ftsEX* variant; all substitutions are located in the FtsX periplasmic domain except K41A and E163Q which are located in the FtsE. OD 600 measurements are shown for the 6 hour time point.

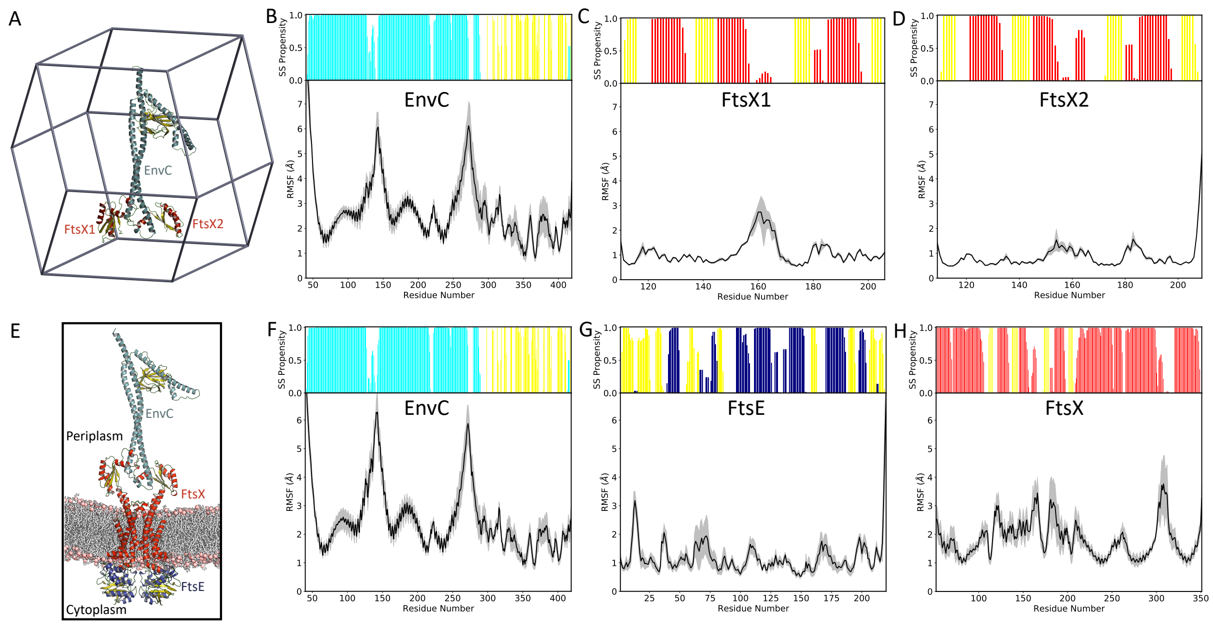

Figure S4: Molecular simulations of the FtsX-EnvC and FtsEX-EnvC complexes. (A) The simulation set-up in a solvated dodecahedron box for the FtsX-EnvC crystal structure. (**B, C** and **D**) Secondary structure and Root Mean Squared Fluctuations (RMSF) plots from three repeats of 100 ns simulations for each protein subunit in the construct. For each plot the secondary structure retention during the simulations is plotted on a 0 to 1 scale, with β-strands coloured yellow and α-helices coloured either cyan (EnvC) or red (FtsX). **(E)** The simulation set-up in a cube for the FtsEX-EnvC model. (**F**, **G** and **H**) Secondary structure and RMSF from three repeats of the membrane solubilised systems. Here the dimeric subunits of FtsE (navy) and FtsX (red) are combined into the same plots.

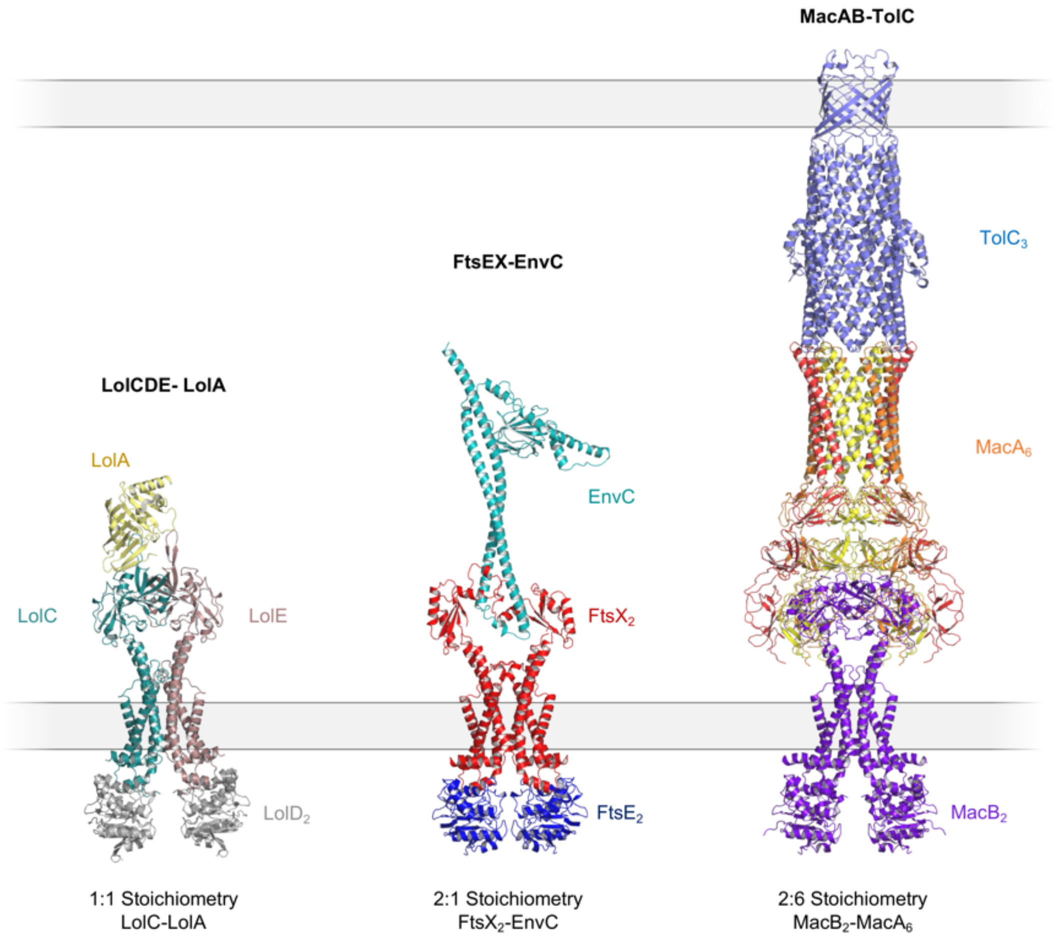

Figure S5: Type VII ABC transporters have structurally-diverse interactions with their periplasmic partners. (*Left*) LolCDE-LolA complex. This model is supported by a crystal structure of LolA bound to the LolC periplasmic domain (6F3Z) (4) and has a 1:1 stoichiometry. (*Centre*) FtsEX-EnvC complex. This model is supported by a structure of EnvC bound to two FtsX periplasmic domains (6TPI) and has a 2:1 stoichiometry. (*Right*) CryoEM structure of the MacAB-TolC complex with 2:6 stoichiometry (5NIL) (7).

Supplemental Methods

DNA Constructs

E. coli ftsEX, *ftsE*, *ftsX*, *envC*, *ftsA* and *amiB* genes (or gene fragments) were amplified by PCR from *E. coli* DH5α genomic DNA. Appropriate restriction sites were engineered into the 5’ ends of each primer to facilitate subsequent cloning into target vectors.

For complementation studies, the *ftsEX* fragment was introduced into pETDuet-1 via BamHI and XhoI restriction sites. This plasmid encodes a histidine tagged FtsE molecule and untagged FtsX. In order to generate various mutants in the same background, we also subcloned the *ftsEX* fragment into pUC18. Point mutations in FtsE and FtsX were then introduced using the Quikchange Protocol (in the pUC/*ftsEX* intermediary) before being cutting out the *ftsEX* fragment with BamH1/XhoI and re-ligating into pETDuet1. To generate the FtsEX construct in which the periplasmic X-lobe was replaced with a pair of glycines [FtsEX(Δ145-171::GG)] we used ‘outward’ PCR with phosphorylated primers, treatment with DpnI, followed by self-ligation. Again, this PCR was performed in the pUC18/*ftsEX* vector and so the mutated form was cut out and re-ligated back into pETDuet1.

For co-expression of EnvC alongside histidine tagged FtsX periplasmic domain, EnvC (coding amino acids 35-419) was ligated into the 3’ MCS of pETDuet via NdeI/XhoI . The periplasmic region of FtsX (110-209) was then cloned into pET21a via NdeI/XhoI to obtain a C-terminal His-tag. The FtsX periplasmic domain fragment, now also coding the tag, was re-amplified from the pET21a/FtsX (110-209) intermediary and cloned into the 5’ MCS of the pETDuet1/-/EnvC vector via NcoI/HindIII sites to produce the expression clone pJC6120.

For clones relating to periplasmic expression of EnvC (and its derivatives), sections of EnvC were PCR amplified and the fragments introduced into pET22b via the NcoI/XhoI sites. We note that residues 1-34 of EnvC encode a *sec*-secretion signal, the DNA for which was excised and replaced by the pelB secretion signal for all our periplasmic expression constructs. Both the *pelB* and natural *envC* sec-signals are proteolytically removed in the periplasm, and thus our pelB:EnvC (35-419) construct produces the same mature EnvC protein as the natural *envC* gene. In addition to the wild type-like pET22b/EnvC(35-419) construct (pJC6260), we also generated constructs lacking the coiled coil domain (pET22b/EnvC(222-419) – pJC6262), or encoding *only* the LytM domain (pET22b/EnvC(278-419) – pJC6263).

For bacterial 2-hybrid experiments, DNA fragments were typically cloned into appropriate vectors of the BACTH system via the BamHI and EcoRI sites, maintaining the open-reading frame. Point mutations were generated by subcloning the ‘parental’ DNA fragment into pUC18, performing Quikchange mutagenesis, and then cutting and re-ligating the mutated fragment back into the Bacterial 2-hybrid vector. Interestingly, ligation of *ftsX* and *ftsA* fragments into pUT18 resulted in many cases of deletions at the ligation points hampering our cloning efforts. This obstacle was overcome by using *E. coli* strain RV308 for the initial transformation of the ligation mix.

In all cases, the presence of the correct sequence in each construct was confirmed by DNA sequencing (Genewiz).

Co-expression and co-purification of EnvC with the FtsX periplasmic domain

E. coli C43 (DE3) transformed with pJC6120 was grown in 2YT medium at 30 °C. At OD_{600nm} 0.6, protein expression was induced by addition of 1 mM IPTG. After 18 hours further growth at 30 °C, cells were harvested by centrifugation (6,000 ×*g*), the cell pellets resuspended in wash buffer (50 mM HEPES pH 7.2, 300 mM NaCl, 30 mM imidazole) and lysed by 2 passages through a Constant Systems Cell Disruptor (30,200 psi). Lysate was centrifuged at 30,000 ×*g* for 30 min at 6 °C to remove cellular debris and the supernatant loaded onto an immobilised Ni-affinity resin (Biorad) pre-equilibrated with the wash buffer. Bound proteins were washed with 25 volumes of wash buffer before elution in 50 mM HEPES pH 7.2, 300 mM NaCl, 250 mM imidazole. Protein samples were buffer exchanged into 20 mM HEPES pH 7.2, 150 mM NaCl and concentrated to ~10 mg/mL using a centrifugal filter (10 kDa nominal molecular weight cut-off) and stored at -80 °C.

Structure determination by X-ray crystallography

The EnvC-FtsX periplasmic domain complex was thawed and then centrifuged for 10 min at 15,000 ×*g* at 6 °C to remove debris immediately prior to setting up crystallisation trials. Crystallisation used the sitting drop vapour diffusion method at 4 °C. Diffraction quality crystals were obtained in the Morpheus crystallisation screen (Molecular Dimensions) in condition 2-22 (Position F10 in the 96-well layout). The crystallisation reagent is composed of 0.02 M D-Glucose, 0.02 M D-Mannose, 0.02 M D-Galactose, 0.02 M L-Fucose, 0.02 M D-Xylose, 0.02 M N-Acetyl-D-Glucosamine, 20 % (v/v) Glycerol, 10 % (w/v) PEG 4,000, 0.1 M Tris/Bicine pH 8.5. Crystals appeared after 1 week and grew to optimum size after 2 weeks. Crystals were harvested in litholoops and plunge-frozen in liquid nitrogen in preparation for data collection at Diamond synchrotron (UK). Data were collected remotely for several crystals, the best diffracting to 2.1 Å resolution. X-ray data were inspected, indexed and integrated using Imosflm (41). Space group determination, scaling and merging used Aimless (42). Phases were determined by molecular replacement using Phaser (45). Molecular replacement searches were conducted with the EnvC LytM domain and periplasmic domain of *M. tuberculosis* FtsX as search probes - initially assuming a 1-to-1 stoichiometry. The EnvC LytM and FtsX periplasmic domain were placed with TFZ scores of 17.8 and 10.6, respectively. After refinement in Refmac (43), phases were further improved using solvent flattening and histogram matching in Parrot (49). Automated model building in Buccaneer (50) helped define the coiled coli domain and resolved several residues in the second FtsX monomer. At this point, it was recognised that the complex had a 2-to-1 stoichiometry and so a new map was calculated using solvent flattening parameters that properly account for the contents of the asymmetric unit. The model was completed using cycles of manual building/rebuilding in Coot (44) and refinement with Refmac (43). We note that the first 40 residues of the FtsX monomer bound to Long helix II are not as well-defined in the electron density as the monomer bound to Long Helix I. Model validation used tools from Coot (44), Procheck (51) and Rampage (48). Further analysis of the structure made use of PISA (26) and Pymol (47).

MIC determinations for Vancomycin, Bacitracin and SDS

Minimum inhibitory concentrations were determined in lysogeny broth (LB) supplemented with 50 µg/mL ampicillin and 1 mM IPTG. Each set of antibiotic/detergent concentrations were generated by 2-fold serial dilutions. Experiments were conducted in 96-well plates and read in a Tecan plate reader after 18 h. Each well had a volume of 200 µL and was seeded with 5 µL of starter culture that was preadjusted to OD_{600} = 0.01 by dilution of seed cultures that were grown to between 0.6 and 1.0 (OD_{600}). MICs are presented as the median of 8 determinations.

Viability of FtsEX variants spotted on antibiotic agar

E. coli strains MR2 (MG1655 *ΔLacI*) and MR10 (MG1655 *ΔLacI ΔFtsEX::Kan*^{R}) were transformed with plasmids carrying either the wild type or variant FtsEX. Cells were grown in LB supplemented to an OD_{600} between 0.6 and 1.0 then diluted to OD_{600} 0.1. Each culture was then diluted a 1000-fold (using 10-fold serial dilutions) and a 3 uL volume spotted onto LB agar containing either Vancomycin or Bacitracin. An antibiotic free LB agar plate was used as a control.

Bacterial 2-hybrid experiments

Bacterial 2-hybrid experiments used the Bacterial Adenylate Cyclase Two Hybrid (BACTH) system (46). 2 ng of each of two complimentary clones were transformed into *E. coli* BTH101 cells. 5 µl of the transformation mix or 5 µl of the mix grown overnight in LB (50 µg/ml ampicillin, 25 µg/ml kanamycin) was spotted onto LB plates containing 50 µg/ml ampicillin, 25 µg/ml kanamycin, 40 µg/ml X-gal and 0.5 mM IPTG and grown at room temperature for ~64 hr. The positive control was a pair of plasmids containing a leucine zipper which dimerises. The negative control was a pair of empty T18 and T25 vectors. Plates were imaged using an Epson scanner and uniformly brightness and contrast adjusted. The presence of blue colonies indicates a positive interaction.

FtsEX complementation experiments

In vivo complementation experiments used *E. coli* strains MR2 (MG1655 *ΔLacI*) and MR10 (MG1655 *ΔLacI ΔFtsEX::Kan*^{R}) with wild type or variant *ftsEX* genes in pETDuet1. A single colony of transformed cells was grown to an OD_{600nm} of 1.0 and then serially diluted using 1-in-10 dilutions. 5 µL of each dilution was spotted onto LB containing 50 µg/mL ampicillin, 1 mM IPTG and with/without 0.1% SDS. Culture was also spotted onto LBON50 (LB with no salt, diluted 2-fold with water) with 50 µg/ml ampicillin and 1 mM IPTG. Plates were incubated overnight at 37 °C. Plates were imaged using an Epson scanner. Images were uniformly contrast-adjusted and converted to greyscale. For liquid broth experiments, growth was monitored in either LBON50, or LB supplemented with 0.1 % SDS, at 37 °C in 96-well plates using a Tecan plate reader. Each well has a volume of 200 µL and was seeded with a 5 uL volume of culture that had previously been grown to OD600 ~1.0 and diluted 100-fold in LB.

Microscopy

E. coli MR10 or MR2 cells carrying pETDuet-based vectors encoding FtsEX variants were grown from glycerol stocks in standard LB supplemented with 50 µg/ml ampicillin and 1mM IPTG at 37 °C. At

approximately OD_{600} = 0.5 cells were diluted 1-in-2 with fresh media and spotted onto an agarose-coated glass slide for immediate observation. Images were collected using the transmitted light channel on a Zeiss 710 point scanning confocal microscope.

Periplasmic expression of EnvC variants

Periplasmic expression of EnvC variants used *E. coli* C43(DE3) strains and were plated on agar plates containing 50 µg/mL ampicillin and either 1 mM or 0 mM IPTG. All strains carry a plasmid defining an ampicillin resistance gene. EnvC constructs all used a pelB leader sequence to ensure equivalent periplasmic expression including the 'full length' EnvC (35-419) construct. EnvC 35-419 was expressed from pJC6260, EnvC (222-419) was expressed from pJC6262, and EnvC (278-419) was expressed from pJC6263. An empty vector (pET21a) was used as the control. Agar plates were imaged using an Epson v370 scanner. Images were uniformly contrast-adjusted and converted to greyscale.

Purification of EnvC variants

Histidine-tagged versions of EnvC variants were expressed in *E. coli* C43 (DE3) from plasmids pJC6 477, pJC6 474 and pJC 276. Cells were initially grown at 30 °C in 2YT media, before induction with 1mM IPTG at 25 °C. Cells were grown overnight, harvested by centrifugation (6,000 g, 6 min, 6 °C) then resuspended in wash buffer (35 mM Imidazole, 150 mM NaCl, 50 mM HEPES 7.2). Resuspended cells were homogenised and small quantity of DNase I and Lysozyme added. Cells were then broken by two passages through the cell disruptor at 30,200 psi. The broken cell suspension was clarified by centrifugation (30,000 g, 15 min, 6 °C) and the cleared lysate incubated in the presence of IMAC resin (BIORAD) for 1 hour with mixing. Protein-bound resin was transferred to a gravity flow column and washed extensively with wash buffer A. Pure proteins were then eluted with buffer B (250 mM Imidazole, 150 mM NaCl, 50 mM HEPES 7.2). All EnvC variants were obtained in high yield and were stable on the bench at room temperature. Purity was assessed by SDS-PAGE.

Co-purification of amidases with the EnvC LytM domain

Histidine-tagged EnvC LytM domain was co-expressed alongside non-tagged forms of either AmiA or AmiB in *E. coli* C43 (DE3) cells grown at 30 C in 2YT media. Co-expression of the EnvC LytM with AmiA used plasmid pTB1011. Co-expression of EnvC LytM with AmiB used plasmid pJC6278. Protein expression was induced with 1mM IPTG for 18 h. Cells were pelleted by centrifugation (6,000 g, 6 min, 6 °C), resuspended in buffer A (35 mM Imidazole, 150 mM NaCl, 50 mM HEPES 7.2), and homogenised. Lysozyme and DNase I were added and cells broken using a cell disruptor (30,200 psi). The lysate was centrifuged to remove debris (30,000 g, 15 min, 6 °C), and the supernatant mixed with Ni-IMAC resin (BIORAD). The mixture was passed through a gravity-flow column to recover protein-bound resin, which was washed with buffer A to remove impurities. Protein complexes were then eluted with Buffer B (250 mM Imidazole, 150 mM NaCl, 50 mM HEPES 7.2). Protein complexes were desalted using PD-10 column (GE Healthcare) and concentrated in buffer GF (150 mM NaCl, 50 mM HEPES 7.5).

Molecular modelling of the FtsEX-EnvC complex

Homology models for FtsE and FtsX were constructed using Phyre webserver (32) based on templates from either ATP-bound (1) or ADP-bound MacB (8) and reassembled in their biologically-relevant oligomeric states by superposing each monomer back onto MacB using Pymol. The ADP-bound/nucleotide free conformation of the FtsEX-EnvC complex was formed by positioning the crystal structure of EnvC bound to the periplasmic domains of FtsX onto the FtsEX homology model before removing the overlap from the homology model. The FtsX periplasmic domains from the crystal structure were then joined to the remaining homology model with minimal rebuilding of the connecting residues in Coot (44). The final model was energy minimised using Refmac (43) and validated by mapping intra- and inter-molecular contacts predicted from co-evolutionary data obtained by Gremlin (33). A model for the ATP-bound form of FtsEX-EnvC was constructed using an assumption that the EnvC N-terminus forms an idealised coiled coil. EnvC residues 35-240 were built as an ideal coiled coil using ccbuilder (52) and the remainder of the LytM domain repositioned accordingly. Each of the FtsX periplasmic domains were then extracted from the crystal structure of the EnvC-FtsX periplasmic domain complex (along with minimal fragments of the EnvC helix to which they were bound) and superposed onto the idealised coiled coil form of EnvC. After a minimal rotation of each domain to relieve a potential clash between each domain, the overlapping segments of EnvC were removed from the homology model and replaced with those taken from the crystal structure. The periplasmic domains were then superposed onto the FtsEX homology model (constructed from ATP-bound MacB) and once again energy minimised after rebuilding the connecting residues.

Molecular dynamics

Simulations were run using GROMACS 2019 (53). The modelled FtsEX-EnvC complex in a membrane was initially configured using the Martini 2.2 coarse-grain (CG) force field and solvated with water and 0.15M NaCl to neutralise the system (54). Membranes were constructed using *insane* with a 4:1 ratio of POPE:POPG lipids (55). An elastic network of 1000 kJ mol^{-1} nm^{-2} was applied between all backbone beads between 0.5 and 1 nm. Electrostatics were described using the reaction field method, with a cut-off of 1.1 nm using the potential shift modifier and the van der Waals interactions were shifted between 0.9-1.1 nm. The system was first energy minimised by steepest descent algorithm to 1000 kJ mol^{-1} nm^{-1} and then simulated for a total of 1 μs. The temperature and pressure were kept constant throughout the simulation at 310 K and 1 bar respectively, with protein, lipids and water/ions coupled individually to a temperature bath by the V-rescale method (56) and a semi-isotropic Parrinello-Rahman barostat (57). The final snapshots from the CG simulations were then converted back to an atomistic description using CG2AT (58). Atomistic simulations for both soluble FtsX-EnvC complex and FtsEX-EnvC model were performed without position restraints for a total of 100 ns, in triplicate. In all cases a 2 fs timestep was used, in an NPT ensemble with V-rescale temperature coupling at 310 K (56) and with a semi-isotropic or isotropic Parrinello-Rahman barostat at 1 bar, used for membrane or soluble constructs, respectively. Protein, water/ions and lipids, if present, were coupled individually (57). Electrostatics were described using PME, with a cut-off of 1.2 nm and the van der Waals interactions were shifted between 1-1.2 nm. The tip3p water model was used, the water bond angles and distances were

constrained by SETTLE (59). H-bonds were constrained using the LINCS algorithm (60). Analysis was performed using MDAnalysis (61) and visualised in PyMOL (47).

Data sharing and materials

Coordinates and structure factors for the structure of EnvC bound to the FtsX periplasmic domain have been deposited with the Protein Data Bank – (PDB entry **6TPI**). Diffraction images underpinning structure determination are available via Zenodo (**DOI 10.5281/zenodo.3574434**). Plasmids available on request.

Legends for Movies

Movie 1: Roving view of the electron density and fitted molecular model for the crystal structure of EnvC bound to the FtsX periplasmic domain. EnvC is shown in *teal* and the FtsX periplasmic domain is shown in *red*. The 2.1 Å $2m|Fo|$-$d|Fc|$ electron density map is shown as a blue mesh contoured at 1 sigma.

Movie 2: Molecular interfaces between FtsX and EnvC. The two interfaces between the FtsX periplasmic domains and EnvC are sequentially examined. Labels for interacting residues are given at **25 s** and **38 s** for the first interface, and **1 min 40 s** and **1min50 s** for the second.

Movie 3: **Structural evidence for an autoinhibition mechanism within EnvC**. This movie shows how the EnvC restraining arm is locked within the amidase binding groove of the LytM domain. Residues implicated in amidase binding (18) are shown in yellow and the restraining arm in pink. This movie complements the static image provided in **Figure 6B**.

Movie 4: Simulations of FtsEX-EnvC. A movie of the MD simulations of the FtsX-EnvC complex and FtsEX-EnvC model embedded in a lipid bilayer. All simulations are run for 100 ns and shown for the three repeats for the two simulation configurations.

Movie 5: **Hypothetical model for the FtsEX-EnvC complex**. The FtsEX-EnvC model is shown in surface representation rotating about an axis perpendicular to the plane of the inner membrane.

Movie 6: **Proposed conformational changes in FtsEX-EnvC based on 3D modelling**. (**0-15 seconds**) Molecular morph between the autoinhibited (ADP-bound) and active (ATP-bound) forms of FtsEX-EnvC. FtsE is shown *blue* with *yellow* β-strands. FtsEX is shown in *red* with yellow β-strands. EnvC is shown in *teal*, LytM domain in *cyan*, restraining arm in *pink*. The EnvC N-terminus (specifically heptads 1-3) are shown in *gold* and heptad 22 is shown in *dark blue*. A diagram of the ATP-binding and hydrolysis cycle is shown to the right. (**15-35 seconds**) Evidence for the proposed conformational change based on the heptads identified in the EnvC N-terminal domain and restraining arm. ADP and ATP-bound forms are shown on left and right,

with molecular morph in centre for which EnvC is coloured by heptad location. Knob residues (position 1 in each heptad) are *red*, and 'hole' residues (position 4 and 5) are *blue*. (**35-45 seconds**) Close-up view showing proposed pairing of heptads 1-3 in the EnvC N-terminus with heptads 22-25 in the restraining arm during the ATP binding and hydrolysis cycle. Co-evolving residue pairs identified by Gremlin (33) (predicting intramolecular contacts) are indicated by cylinders. (**45-59 seconds**) Close-up view of the FtsX periplasmic domains as they squeeze the EnvC N-terminal domain. See discussion for full details of the proposed mechanism.

Supplemental References

48. Lovell SC, et al. (2003) Structure validation by C alpha geometry: phi,psi and C beta deviation. *Proteins-Structure Funct Genet* 50(3):437–450.
49. Cowtan K (2010) Recent developments in classical density modification. *Acta Crystallogr Sect D Biol Crystallogr* 66:470–478.
50. Cowtan K (2006) The Buccaneer software for automated model building. 1. Tracing protein chains. *Acta Crystallogr Sect D Biol Crystallogr* 62:1002–1011.
51. Laskowski R a., MacArthur MW, Moss DS, Thornton JM (1993) PROCHECK: a program to check the stereochemical quality of protein structures. *J Appl Crystallogr* 26(2):283–291.
52. Wood CW, Woolfson DN (2018) CCBuilder 2.0: Powerful and accessible coiled-coil modeling. *Protein Sci* 27(1):103–111.
53. Abraham MJ, et al. (2015) Gromacs: High performance molecular simulations through multi-level parallelism from laptops to supercomputers. *SoftwareX* 1–2:19–25.
54. De Jong DH, et al. (2013) Improved parameters for the martini coarse-grained protein force field. *J Chem Theory Comput* 9(1):687–697.
55. Wassenaar TA, Ingólfsson HI, Böckmann RA, Tieleman DP, Marrink SJ (2015) Computational lipidomics with insane: A versatile tool for generating custom membranes for molecular simulations. *J Chem Theory Comput* 11(5):2144–2155.
56. Bussi G, Donadio D, Parrinello M (2007) Canonical sampling through velocity rescaling. *J Chem Phys* 126(1). doi:10.1063/1.2408420.
57. Parrinello M, Rahman A (1981) Polymorphic transitions in single crystals: A new molecular dynamics method. *J Appl Phys* 52(12):7182–7190.
58. Stansfeld PJ, Sansom MSP (2011) From coarse grained to atomistic: A serial multiscale approach to membrane protein simulations. *J Chem Theory Comput* 7(4):1157–1166.
59. Miyamoto S, Kollman PA (1992) Settle: An analytical version of the SHAKE and RATTLE algorithm for rigid water models. *J Comput Chem* 13(8):952–962.
60. Hess B, Bekker H, Berendsen HJC, Fraaije JGEM (1997) LINCS: A Linear Constraint Solver for molecular simulations. *J Comput Chem* 18(12):1463–1472.
61. Michaud-Agrawal N, Denning EJ, Woolf TB, Beckstein O (2011) MDAnalysis: A toolkit for the analysis of molecular dynamics simulations. *J Comput Chem* 32(10):2319–2327.
